# Molecular Determinants of Optical Modulation in ssDNA–Carbon Nanotube Biosensors

**DOI:** 10.1021/acsnano.4c13814

**Published:** 2025-01-16

**Authors:** Andrew T. Krasley, Sayantani Chakraborty, Lela Vuković, Abraham G. Beyene

**Affiliations:** 1 Janelia Research Campus, Howard Hughes Medical Institute, Ashburn, Virginia 20147, United States; 2 Department of Chemistry and Biochemistry, University of Texas at El Paso, El Paso, Texas 79968, United States; 3 Computational Science Program and Bioinformatics Program, University of Texas at El Paso, El Paso, Texas 79968, United States

**Keywords:** single-walled carbon nanotubes, dopamine, biosensors, fluorescence, DNA, screening, molecular dynamics

## Abstract

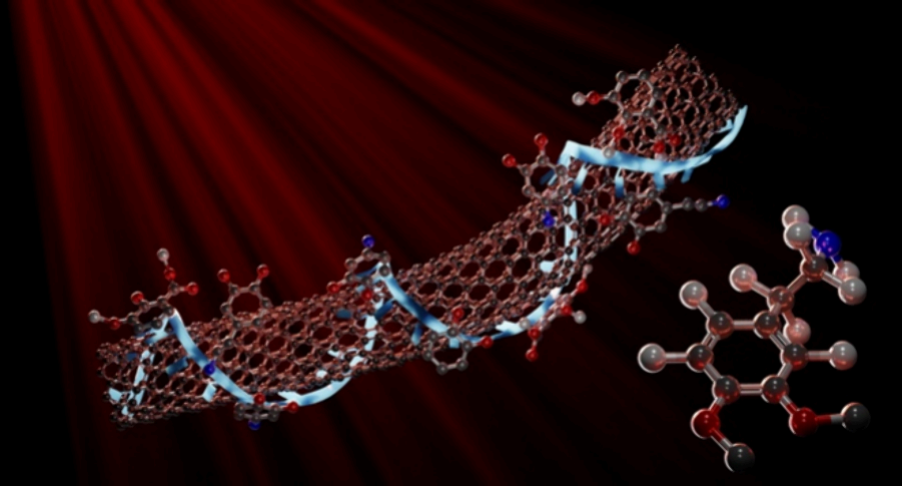

Most traditional optical biosensors operate through molecular recognition, where ligand binding causes conformational changes that lead to optical perturbations in the emitting motif. Optical sensors developed from single-stranded DNA-functionalized single-walled carbon nanotubes (ssDNA–SWCNTs) have started to make useful contributions to biological research. However, the mechanisms underlying their function have remained poorly understood. In this study, we combine experimental and computational approaches to show that ligand binding alone is not sufficient for optical modulation in this class of synthetic biosensors. Instead, the optical response that occurs after ligand binding is highly dependent on the chemical properties of the ligands, resembling mechanisms seen in activity-based biosensors. Specifically, we show that in ssDNA–SWCNT catecholamine sensors, the optical response correlates positively with the electron density on the aryl motif, even among ligands with similar ligand binding affinities. Importantly, despite the strong correlations with electrochemical properties, we find that catechol oxidation itself is not necessary to drive the sensor optical response. We discuss how these findings could serve as a framework for tuning the performance of existing sensors and guiding the development of new biosensors of this class.

The photoluminescence properties of single-walled carbon nanotubes (SWCNTs), which originate from quantum-confined surface excitons, have been exploited for a variety of biological applications, including fluorescence imaging,^1^ single-particle tracking,^2−5^ and biosensing.^1,6,7^ These applications take advantage of the nanotube’s unique photophysical traits, such as photoluminescence in the near-infrared and shortwave infrared regions of the spectrum, as well as their nonblinking and photostable emission. In biosensing, the excitonic fluorescence of SWCNTs and their distinctive single-atom-thick geometry are exploited to translate molecular recognition events into detectable optoelectronic signals. The optoelectronic properties of SWCNTs, and similar shell-like nanomaterials, are highly sensitive to physicochemical perturbations that occur on or near the surface, enabling detection of local changes with single-molecule sensitivity. This has been successfully demonstrated in functionalized SWCNTs.^8−10^

Biosensing applications of SWCNTs require functionalization with moieties that tailor the pristine surface of the nanomaterials, creating configurations that are ideal for analyte binding. Among the various strategies for developing SWCNT-based biosensors, noncovalent functionalization with amphiphilic biopolymers, particularly oligonucleotides (e.g., single-stranded DNA (ssDNA)), remains a predominant strategy.^1,11−16^ This approach enables versatile patterning of the nanotube surface with chemically rich and structurally diverse oligonucleotide motifs. This strategy has enabled successful applications of ssDNA–SWCNT hybrids in diverse fields, including nanotube-based device manufacturing,^17,18^ chirality sorting,^19,20^ and SWCNT lattice remodeling.^21^ The conjugation of ssDNA to the surface of nanotubes through noncovalent self-assembly sculpts specific analyte binding pockets that are absent on nonfunctionalized surfaces, enabling their use in biosensing applications.^1,11−16^

Despite several successful applications, a coherent strategy for developing biosensors from ssDNA–SWCNT bio–nano hybrids remains elusive. This challenge stems in part from a lack of understanding of how analyte binding in ssDNA–nanotube bio–nano conjugates modulates the optical properties of the nanotubes. The diversity of nanotube surface topologies that can be engineered with ssDNA sequences is vast and depends on the oligonucleotide length and sequence chemistry. Matching this broad chemical space to potential analytes through screening approaches is an arduous task, and success with this approach has been limited.^11,22,23^ However, recent studies have demonstrated that machine learning approaches hold promise for predicting new ssDNA sequences for sensing small molecular analytes.^24−26^ An alternative approach is rational design, where a mechanistic understanding of how ssDNA–SWCNT biosensors function guides sensor development. Such an understanding could streamline the development of biosensors by informing the selection or chemical modification of ssDNA sequences and the design of nanotube functionalization strategies, ultimately leading to more effective and predictable sensor development and performance.

To better understand the mechanisms of fluorescence modulation in ssDNA–SWCNTs, we performed a structure–activity relationship study on a class of sensors for catechol (benzene-1,2-diol)-bearing small molecules. Previous studies have shown that (GT)_*N*_–SWCNT (*N* = 6–15) conjugates undergo a strong fluorescence turn-on in response to catecholamines, with reported affinities in the nanomolar to single micromolar range.^11,27,28^ These sensors have enabled significant advancement in the field of catecholamine biology, including the study of dopamine (4-(2-aminoethyl)benzene-1,2-diol), in cell cultures^28−30^ and tissues.^27^ Nanotube-based catecholamine sensors are notable for their robustness, intensiometric readout, high signal-to-noise ratio, and rapid and reversible responses—attributes highly valued in biological applications. We propose that this class of sensors can serve as a model system from which mechanistic insights benefiting the broader field may emerge. By focusing on these well-characterized systems, we aimed to elucidate the fundamental principles governing the interaction between ssDNA-functionalized SWCNTs and their analytes, potentially paving the way for the rational design of new biosensors.

In pursuit of this goal, we combined experimental and computational approaches to better understand how compounds bearing the catechol motif modulate the fluorescence of (GT)_6_–SWCNT conjugates. Experimentally, we observed that optical modulation in these sensors is strongly influenced by certain electrochemical properties of catechols. Manipulating electron densities on the aryl motif of catechols sensitively alters the fluorescence turn-on response, with higher electron densities correlating positively with a stronger turn-on response. Correlational analysis with reduction potentials also reflected this trend, where electron-rich catechols, which oxidize more easily, elicited stronger fluorescence turn-on responses. Notably, however, no oxidative products were generated during the molecular recognition process, as implied by the correlation between the optical response and reduction potential. This suggested that transient perturbative phenomena, rather than permanent charge transfer, are responsible for the optical modulation observed in these sensors. To rationalize our experimental observations, we employed molecular dynamics (MD) simulations. These simulations provided insights into analyte–sensor interactions, which, when combined with experimental data, allowed us to identify key molecular parameters that collectively define a “perturbation cross section” for catechol-bearing ligands. Our work suggests that ligand binding and analyte electrochemical properties play a concerted role in modulating optical responses in ssDNA–SWCNT biosensors.

## Results

**Figure 1:**
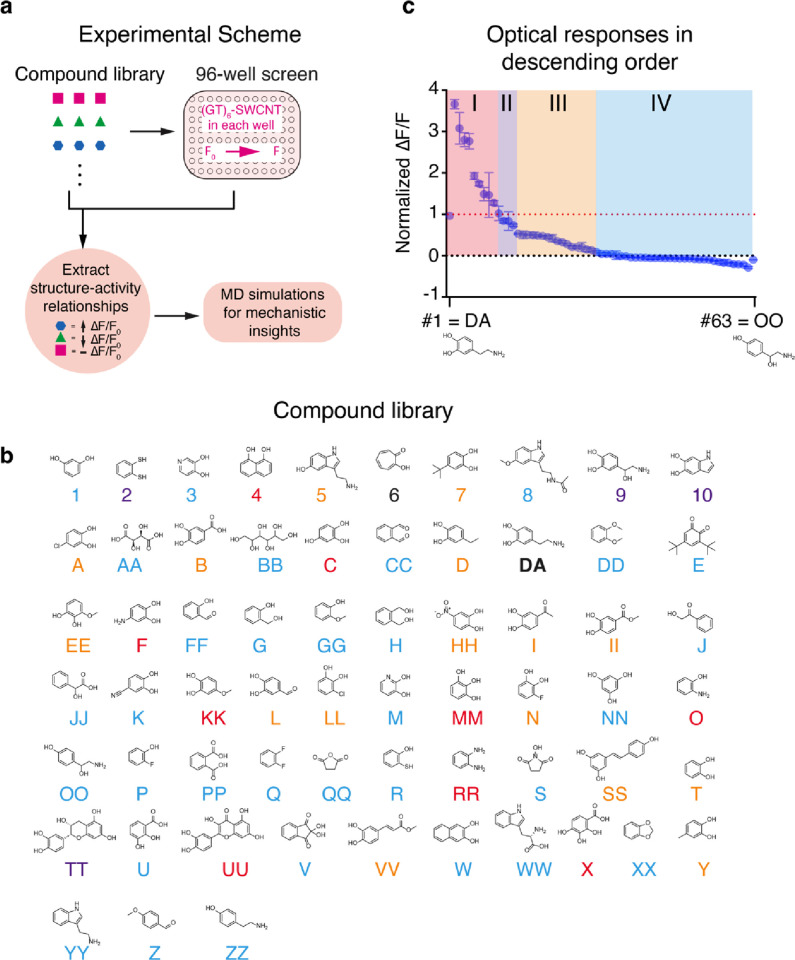
Experimental schematic and screening library. (a) Study workflow. (b) Library of the 63 compounds screened; labels are color coded to match the optical response categories shown in panel (c). (c) Normalized (mean) fluorescence responses (normalized to dopamine = 1.00) in descending order. Group I analytes have high responses (≥1.00), group II have intermediate responses (0.75–1.00), group III have low responses (0.15–0.50), and group IV elicit no response (<0.10). See Table S1 for a full list of Δ*F* and *F* mean values and standard deviations. Under the experimental conditions used to measure optical responses, species with primary amine groups (e.g., **DA**, **F**, **5**, **9**, **RR**, and **O**) may exist in their protonated state; however, their positive charge designations are omitted here for clarity.

To investigate optical responses in (GT)_6_–SWCNT conjugates, we generated a library of small molecules, using dopamine (**DA**) as our principal compound. The library was designed with variations in the truncation, extension, and substitution patterns around the aryl group. The library included conjugated and unconjugated systems as well as aryl groups with bulky substituents to assess steric effects. Electron-donating and electron-withdrawing substituents, along with hydrogen bond donors and acceptors, were installed at various positions to explore electronic effects and binding interactions (Figure 1). We measured the fluorescence modulation caused by each analyte in solution-phase experiments by recording the emission intensity before and after the addition of 10 μM of each analyte and reported the relative change in intensity from integrated spectra (Δ*F*/*F*) (Figure S1). To enable comparison across replicates, we normalized the responses to the modulation measured for dopamine under the same experimental conditions. The screening results showed that a majority of the screened analytes produced no optical responses (Figure 1c IV, 53%). The responses generated by the remaining analytes varied widely and included optical modulations that are stronger than that produced by dopamine (Figure 1c I, 15%), comparable to that of dopamine (Figure 1c II, 6%), and weaker than that of dopamine (Figure 1c III, 26%).

Closer examination of the screening results reveals a key requirement to elicit optical response: *ortho*-hydrogen bond donors installed on π-conjugated scaffolds. Noncatechols can be tolerated if they satisfy these two criteria (e.g., **RR** and **O**). Changing the position of the hydrogen bond donors from *ortho* (**T**) to *meta* (**1**) leads to the loss of optical response. Interestingly, compound **4** elicited a response, even though the hydroxys are closer than the van der Waals radii on *ortho*-substituted aryls. This suggests that conjugated systems with hydrogen bond donors less than ∼3.1 Å^31^ apart could also be effective sensor substrates. Loss in response also occurs if the hydrogen bond donors are unconjugated (e.g., **BB**, **G**, and **H**) or replaced with an acceptor (e.g., **FF**, **6**, **GG**, **P**, and **Q**). Sterically bulky compounds meeting these criteria were well tolerated in producing responses (e.g., **4**, **TT**, **UU**, and **W**). This suggests that the binding pocket of interactions is likely to be shallow or is at least very accessible. In later sections, we rationalize these experimental observations by using MD simulations that enable us to visualize how molecular structure and charge influence analyte–sensor binding.

As expected, most optical modulations were generated by compounds bearing the catechol motif, whereas other molecules generated modest or no responses. Interestingly, the catechol motif was not a guarantee of the presence of a fluorescence modulation, and even within the catechol family of molecules, optical responses varied widely (e.g., **MM** > **DA**^+^ > **T** > **U**, Figure 1). This suggests that while the previously described heuristics serve as useful qualitative descriptors, they fail to fully account for the observed trends in the optical responses. Additionally, because all optical measurements used in this study were recorded at 10 μM analyte concentrations, we explored whether differences in ligand binding affinity might explain the variability in the optical modulation. To investigate this, we measured optical responses for a smaller subset of analytes over a concentration range spanning 6 orders of magnitude (10^–8^–10^–2^ M). Notably, the trends in optical responses remained consistent across the concentration range we examined (Figure S2). This result, along with insights from MD simulations presented in subsequent sections, strongly suggests that binding affinity differences are not the primary driver of the observed variability in the optical response. Consequently, we turned our attention to other molecular properties that might provide a more robust quantitative explanation for the observed variability in the optical responses.

**Figure 2:**
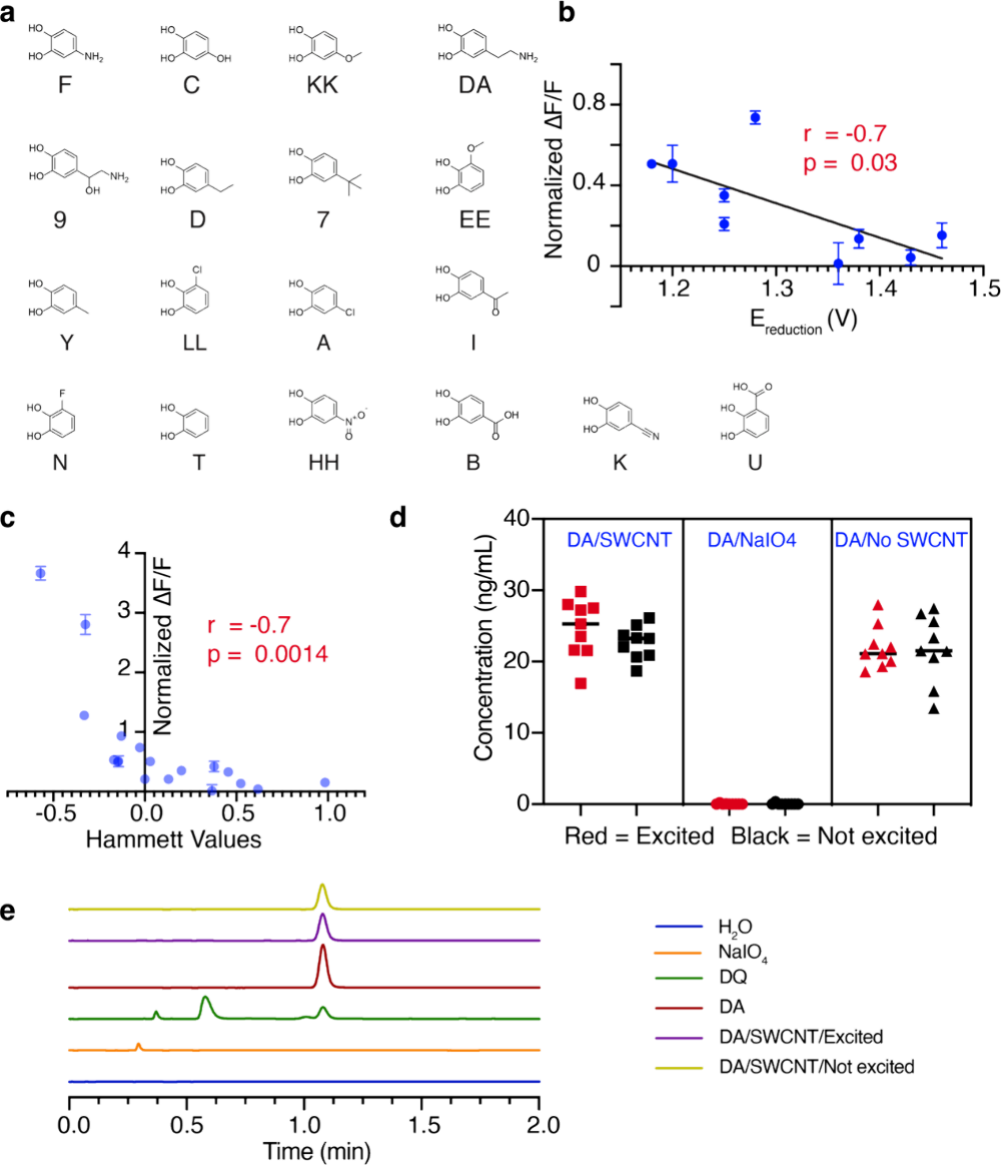
Molecular correlates of the optical response. (a) Subset of 18 compounds from the 63 screened, used for comparative analysis. Positive charge designations on **DA**, **F**, and **9** are omitted for clarity. (b) Normalized fluorescence responses (mean, **DA** = 1.00) vs reduction potentials from Pelizzetti and Mentasti^32^ for nine compounds. Compounds that more readily underwent oxidation produced larger changes in fluorescence. See Table S2 for Δ*F*/*F* values and reduction potentials. The Pearson statistic was evaluated to assess correlation. (c) Normalized fluorescence response (mean, **DA** = 1.00) vs Hammett values for the 18-compound subset in panel (a), demonstrating that more electron-donating substituents produced larger changes in fluorescence. See Table S3 for the Δ*F*/*F* and Hammett values. The Pearson statistic was evaluated to assess correlation. (d) Dopamine ELISA showing no difference in amounts of dopamine between samples exposed to 658 nm light (104.8 mW, 60 min, excited) and those that were unexposed (not excited). As a control, dopamine was oxidized to dopaquinone (DQ) with NaIO_4_ to demonstrate that dopamine depletion (by oxidation to quinone) can be detected as a loss of signal by the assay (DA/NaIO_4_). As another control, dopamine in 0.1 M NaCl without SWCNTs in solution was exposed to laser to show that little oxidation occurs under these conditions (DA/no SWCNT). See Figure S5 for assay validation and statistics. (e) High-performance liquid chromatography (HPLC) analysis of exposed (658 nm, 104.8 mW, 60 min, excited) and unexposed (not excited) samples along with controls showing that only dopamine was detected in both samples with no detection of dopaquinone or any other oxidized products (blue: H_2_O; orange: NaIO_4_; green: dopaquinone (dopamine oxidized with a substoichiometric amount of NaIO_4_); red: dopamine; purple: 10 ppm of SWCNT + 200 μM dopamine exposed to 658 nm light for 1 h; yellow: 10 ppm of SWCNT + 200 μM dopamine exposed to no light for 1 h). Higher concentrations of dopamine were used in HPLC experiments based on the LOD of the instrument. See Figure S6 for details and instrument exports and Methods for details of the experiments.

Toward this goal, we explored the molecular correlates of optical modulation by assessing if certain physicochemical parameters of these molecules correlated with optical responses. To facilitate comparison and minimize contributions that might arise from significant differences in molecular structure and steric effects, we selected a subset of 18 compounds, each bearing a catechol motif with simple substituents at different positions on the aryl ring (Figure 2a). We then investigated the correlation between 12 different cheminformatic parameters of these molecules and the optical modulations generated by each. These properties included the strength of the dipole moment, polarizability, LogP, and van der Waals surface area, among others. The analysis demonstrated a general lack of correlation between the experimentally measured optical responses and all 12 of the physicochemical attributes examined (Figures S3 and S4).

The electrochemical properties of catecholamines have traditionally been exploited for their characterization and quantification using techniques such as amperometry, cyclic voltammetry, and liquid chromatography with tandem mass spectrometry.^33−35^ Similarly, optical modulations in some SWCNT-based sensors have been shown to be driven by the redox activities of their target analytes, with electrochemical mechanisms posited as the basis for fluorescence modulation.^9,36,37^ Given our observation of a wide range of optical responses, even in compounds bearing simple catechol motifs, we set out to conduct a deeper exploration of whether the observed optical trends correlated with the electrochemical properties of the screened molecules. Specifically, we wanted to know if experimental optical responses correlated with reduction potentials in our selected subset of catechol compounds.

The standard reduction potentials of substituted catechols have previously been determined through kinetic studies of their one- and two-electron oxidation by tris(1,10-phenanthroline) iron(III).^32,38^ From our screening library of 63 compounds, nine compounds overlapped with a library of 15 analytes for which standard reduction potentials were experimentally determined by Pelizzetti and Mentasti.^32^ For these nine ligands, our analysis unveiled a robust correlation between the reduction potentials of the substituted catechols and their corresponding optical responses (Figure 2b). Specifically, compounds that underwent facile oxidation elicited more pronounced optical responses than compounds that were more difficult to oxidize (Figure 2b). This finding suggested that electrochemical properties are significant correlates of optical response, although this observation was based on a relatively small subset of our library. To validate and extend these findings, we aimed to explore whether this observation holds true across a broader range of molecules in our screening library.

Expanding on the work of Pelizzetti and Mentasti, Yamabe et al. demonstrated that the reduction potentials of substituted benzene diols are correlated with the electron-donating or electron-withdrawing character of the substituents (*X*) on the aryl group.^32,38^ Specifically, they showed that the HOMO of substituted catechols is composed of two types of interactions between the molecular orbitals (MOs) of the parent compound (benzene-1,2-diol, *P*) and the substituent (*X*). The first interaction, known as HOMO_*P*_–HOMO*_X_*, occurs between the HOMO of the parent molecule (HOMO_*P*_) and the HOMO of the substituent (HOMO_*X*_). The second interaction involves HOMO_*P*_ and the lowest unoccupied molecular orbital (LUMO) of the substituent (LUMO_*X*_), known as the HOMO_*P*_–LUMO_*X*_ interaction. The electron-donating or electron-withdrawing nature of substituent *X* determines which combination of MOs, HOMO_*P*_–HOMO_*X*_ or HOMO_*P*_–LUMO_*X*_, predominates in controlling the HOMO of the substituted compound. Yamabe et al. showed that for electron-donating groups, HOMO_*P*_ and HOMO_*X*_ have a strong orbital interaction, and the resultant energy splitting opens a large energy gap that raises the energy level of the HOMO of the overall molecule, making it relatively easier to oxidize. In contrast, for electron-withdrawing groups, HOMO_*P*_–HOMO_*X*_ interactions are insignificant, and HOMO_*P*_–LUMO_*X*_ interactions are important for setting the HOMO level of the overall molecule, lowering the HOMO level of the substituted molecule relative to that of the parent molecule (i.e., benzene-1,2-diol), thus making the molecule more difficult to oxidize. Using this theoretical framework, Yamabe et al. showed a strong linear correlation between the experimentally determined reduction potentials of substituted benzene-1,2-diols and computationally determined HOMO levels (*e*_HOMO_). This correlation highlights that the electron-donating or electron-withdrawing character of substituents and computationally obtained *e*_HOMO_ levels are excellent predictors of reduction potentials for substituted benzene-1,2-diols. This study therefore enabled extending the correlation analysis between optical response and electrochemical properties to a broader range of the screened analytes, where reduction potentials had not been experimentally determined but could be reasonably approximated with substituent inductive constants or computationally determined *e*_HOMO_ values.

A key finding from the study by Yamabe et al. is that electron-donating groups raise the *e*_HOMO_ values of benzene-1,2-diols, making them easier to oxidize. Accordingly, we first extended our correlation analysis between optical modulation and reduction potentials to the 18-compound subset library (Figure 2a). Here, we used the Hammett constant of each substituent as a correlate for the reduction potential. The analysis unveiled a robust correlation between the experimentally measured optical modulations and the Hammett values of each substituent. Specifically, electron-donating substituents produced stronger optical modulations (Figure 2c and Figures S7 and S8a). Moreover, for the same substituent *X*, Yamabe et al. demonstrated that the atomic orbital coefficients at the *para*-positions (4 or 5) are larger than those at the *meta*-/*ortho*-positions (3 or 5), leading to more robust orbital interactions that strongly modulate HOMO levels. For instance, placement of a hydroxy group at the *para*-position of benzene-1,2-diol (**C**) significantly increases the electron density in the aryl compared to placement at the *meta*-/*ortho*-position (**MM**), making the molecule more easily oxidizable (Figure S7a). Notably, the optical responses we measured correlated well with such subtle differences between isomers of the same compound (Figure S7a,b) and in compounds that differed in just one functional group (Figure S7c,d).

Because Yamabe et al.’s framework ultimately implicates *e*_HOMO_ values as correlates for reduction potentials, it allowed us to extend this analysis to molecules in our library lacking a simple catechol motif or for which Hammett values could not be found but for which overall HOMO values can be computed. Consequently, correlations between optical modulations and computationally determined *e*_HOMO_ levels were examined for various subsets of the screened analytes. Here, too, a robust correlation was observed between optical responses and HOMO levels for the 18-compound sublibrary (Figure S8b), as well as various other subsets (Figure S9a,b) and the entire library of compounds (Figure S9c). Importantly, while correlation between HOMO and optical responses was evident, high *e*_HOMO_ levels did not guarantee an optical response (Figure S9c,d). This suggests that additional factors are at play beyond electron density alone. Consistent with our earlier heuristic description, these results show that at least two vicinal hydrogen-bond-donating groups are necessary for optical modulation in addition to the observed correlations with electron densities on the aryl group (Figure S9e). In most cases, these groups are *ortho* to each other but they can be connected through extended conjugation as well (e.g., **4**, Figure S9e).

Next, we investigated whether oxidized catechol products could be detected when these compounds were exposed to ssDNA–SWCNT conjugates, as suggested by the correlations presented in the foregoing analysis. We first used an enzyme-linked immunosorbent assay (ELISA) that can sensitively quantify the concentration of dopamine at picomolar concentrations but is otherwise insensitive to quinones, the oxidative product of catechols (Figure S5). Oxidation of dopamine with sodium periodate^39^ induced rapid depletion of the starting material, which we verified with the ELISA (Figure 2d). We then quantified the level of oxidation of dopamine in solutions that had been exposed to ssDNA–SWCNT conjugates for various durations and excitation laser intensities. Surprisingly, no oxidation product was detected using this assay, indicating minimal oxidation of dopamine in the starting material (Figure 2d). Similarly, high-performance liquid chromatography (HPLC) detection of quinones showed depletion of the starting material in periodate controls (Figure 2e, dopaquinone (DQ)) but not in experimental solutions (Figure 2e, DA/SWCNT/excited and DA/SWCNT/not excited).

Oxidation of benzene diols proceeds through a one-electron abstraction to form a semiquinone radical,^40^ and we reasoned that if a radical is formed during the process of generating optical modulations, highly reducing reagents should attenuate or eliminate these optical responses. Similarly, if electron transfer reactions occur, then dissolved molecular oxygen could act as an electron sink or play a role as an intermediate in a putative electrochemical reaction, generating reactive oxygen species. We observed no attenuation in optical response in the presence of reducing reagents and reactive oxygen scavengers, consistent with the absence of oxidation from ELISA and HPLC measurements (Figure S10). In summary, our findings indicate that redox reactions involving dissolved molecular oxygen or single-electron transfers that generate radicals are unlikely to be present. Previous studies have similarly shown that reactive oxygen species are unlikely to be involved during catecholamine molecular recognition.^11^ We therefore conclude that although optical responses showed a strong correlation with the electrochemical properties of benzene-1,2-diols, the evidence indicates that the analytes themselves do not undergo oxidation during the process of optical modulation of ssDNA–SWCNT conjugates.

**Figure 3:**
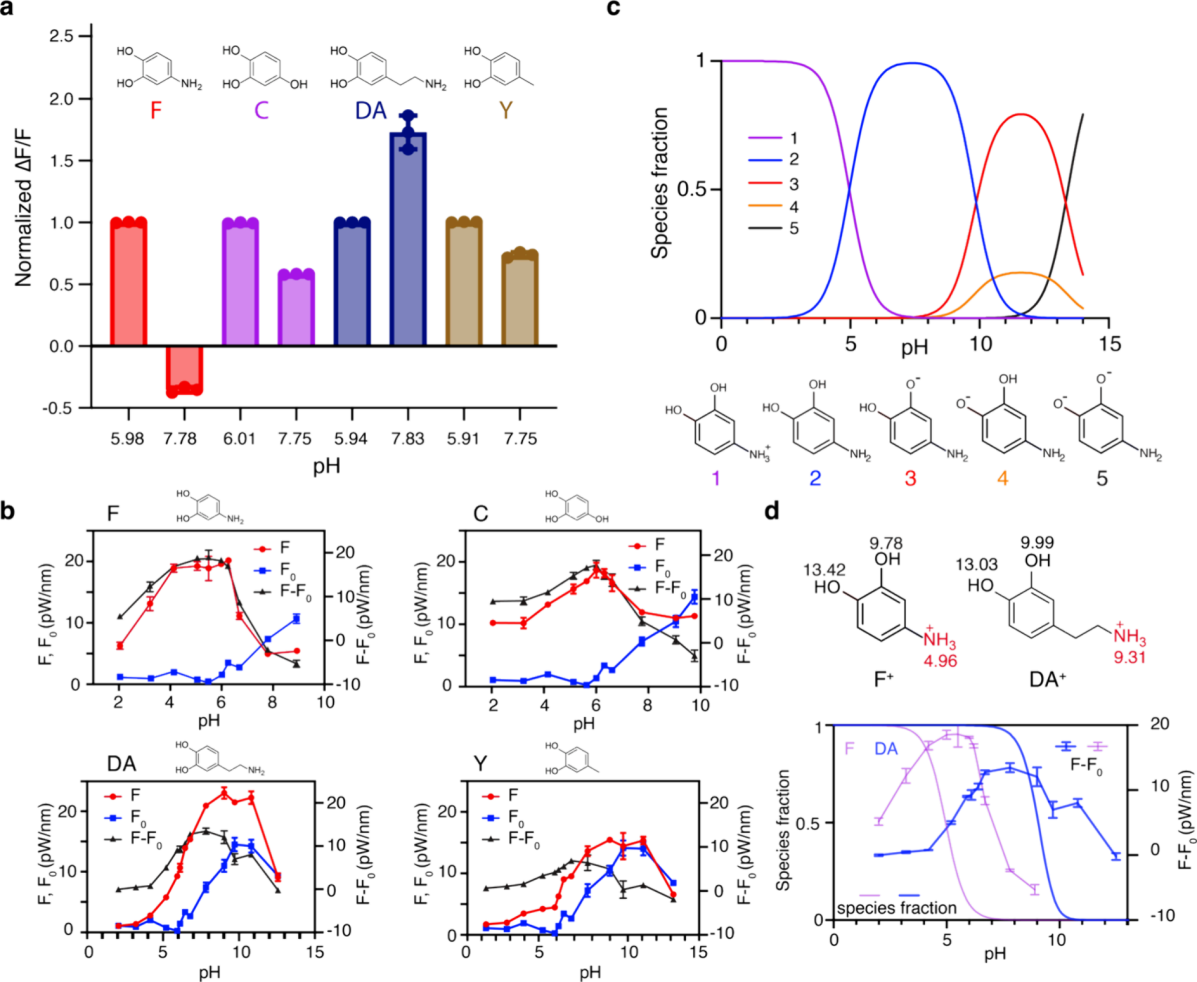
Effect of solution pH on Δ*F*/*F* for several compounds. (a) Four example compounds that exhibit different responses at low (pH ∼ 6) and high (pH ∼ 8) solution pH; Δ*F*/*F* values (mean) were normalized against the response at pH ∼ 6. Positive charge designations on **F** and **DA** are omitted for the sake of clarity. (b) Responses in compounds in panel (a) measured across a range of pH. For analytes where the fluorescent response was less than the baseline (i.e., *F* – *F*_0_ ≤ 0), higher pH values were not further explored. (c) p*K*_A_ model of **F** showing proportions of microspecies at different pH values. (d) Comparison of **F** (purple with error bars) and **DA** (blue with error bars) Δ*F* (*F* – *F*_0_) values as a function of pH, juxtaposed against microspecies transition profiles from ammonium to amine (solid lines without error bars in the same respective colors). Note the p*K*_A_ values for the ammonium in **F**^+^ and **DA**^+^ shown as a red numerical text adjacent to the functional group.

In our experiments, we observed that solution pH, a key experimental variable, could have a significant and yet underappreciated impact on the magnitude of the measured optical modulations. The influence of pH on SWCNT optical properties is well documented, with a general increase in brightness noted for most SWCNT solutions as pH increases.^41,42^ However, the effect of pH on analyte-induced optical modulation is not well understood. We found that changing the solution pH by just two units could dramatically attenuate the optical response for some analytes (Figure 3a). This effect is particularly pronounced for analytes with substituents whose p*K*_A_ values allow deprotonation within this range.

To probe pH effects more systematically, we measured the optical responses of a subset of analytes across a pH range of 2 to 13 (Figure 3b and Figure S11). The (GT)_6_–SWCNT suspensions remained stable through this range, except at pH = 13, where sensor instability was evident (Figure S12). At high pH levels (>8), optical responses diminished significantly for all analytes. This reduction is partly attributed to an increase in nanotube fluorescence (i.e., brightness) with a rising pH, which reduces the dynamic range of the optical response. Specifically, as baseline fluorescence (*F*_0_) increases, the relative change in fluorescence (Δ*F*/*F*_0_) decreases, effectively limiting the sensor’s response. In this context, pH sets the baseline brightness (*F*_0_), while ligand addition determines the final brightness (*F*). However, certain benzene-1,2-diol derivatives in our screen induced a maximum brightness that exceeded the fluorescence at a high pH. This indicates that pH-induced brightness saturation alone does not account for the observed pH-dependent sensor behavior (Figure 3b and Figure S11). Indeed, the diminution of Δ*F*/*F* as a function of pH varied significantly among analytes. For example, some analytes, like **F**^+^, exhibited a rapid decline in optical response with increasing pH (e.g., **F**, Figure 3b), while others, like dopamine (**DA**^+^), showed broader pH tolerance (e.g., **DA**, Figure 3b). Given that the same ssDNA–SWCNT complex was used for all analytes, the pH dependencies are unlikely to stem from changes in the photophysical properties of the ssDNA–SWCNT conjugates. While intrinsic sensor properties, such as DNA conformational changes or base protonation/deprotonation, might affect responses at extreme pH levels (e.g., <3 or >11), the pronounced differences observed within the pH range of 5 to 9 are likely driven by analyte-specific effects.

Importantly, these pH-dependent trends correlated with the predicted deprotonation of the analytes based on the p*K*_A_ values of their substituents. For example, the p*K*_A_ of the charged amine (−NH_3_^+^) in **F**^+^ is 4.96, whereas for **DA**^+^, it is 9.31, allowing **DA**^+^ to maintain a broader pH tolerance before deprotonation (Figure 3c,d and Figure S13). This suggests that the deprotonated species interact with ssDNA–SWCNT conjugates differently from their protonated counterparts (e.g., **F** vs **F**^+^ and **DA** vs **DA**^+^). Consequently, optical modulations in ssDNA–SWCNT conjugates by benzene-1,2-diol derivatives reflect a sensitivity to molecular charge in addition to correlations with electrochemical properties, such as *e*_HOMO_. Taken together, these findings emphasize the critical role of molecular structure and charge in mediating analyte–ssDNA–SWCNT interactions and in governing optical responses in molecules with favorable electrochemical profiles. Importantly, our observations were consistent across different (GT)_*N*_–SWCNT sensors, including (GT)_15_–SWCNTs tested with a subset of compounds (Figure S14a,b,e). The trends also persisted across SWCNTs enriched for specific chiral (*n*,*m*) species, indicating that our results are conserved across diverse nanotube species (Figure S14a,c,d,f).

We next employed MD simulations to rationalize our experimental findings and gain detailed atomic and molecular insight into molecular interactions in ssDNA–SWCNT conjugates. We focused on a subset of 14 analytes to study using MD simulations (Methods). Dopamine (**DA**^+^) was selected as the key model analyte for our computational studies, which is consistent with our experimental approach. The remaining analytes spanned the full spectrum of experimentally observed optical responses. Specifically, the simulated molecules included analytes **F**^+^, **C**, and **O**, which elicited stronger fluorescence responses than **DA**^+^, and analytes **T** and **1**, which showed weaker responses. Analyte **Y**, which had a response roughly similar to that of dopamine, was also included. To explore pH effects, we modeled additional molecules in their dominant protonation states at selected pH values (Figure S15). These included **F**^+^ (at pH = 5), **F** (at pH = 7), **RR** (at pH = 7), **RR**^+^ (at pH = 3), **YY**^+^ (at pH = 7), **O** (at pH = 7), **O**^+^ (at pH = 3), **5**^+^ (at pH = 7), and **C**^–^ (at pH = 11). We determined molecular charge using Chemaxon Chemicalize modeling of p*K*_A_, matched to the pH conditions used during experimental measurements.^43^ Each simulated system contained six molecules of the selected analyte, which were allowed to diffuse freely and interact with a 4 nm segment of (9,4)-SWCNT chirality wrapped by three strands of (GT)_6_ ssDNA oligonucleotides, immersed in 0.10 M aqueous NaCl solution (Methods). The systems were simulated for 6 μs to observe multiple binding and unbinding events of analyte molecules on the ssDNA–SWCNT conjugate surface.

**Figure 4:**
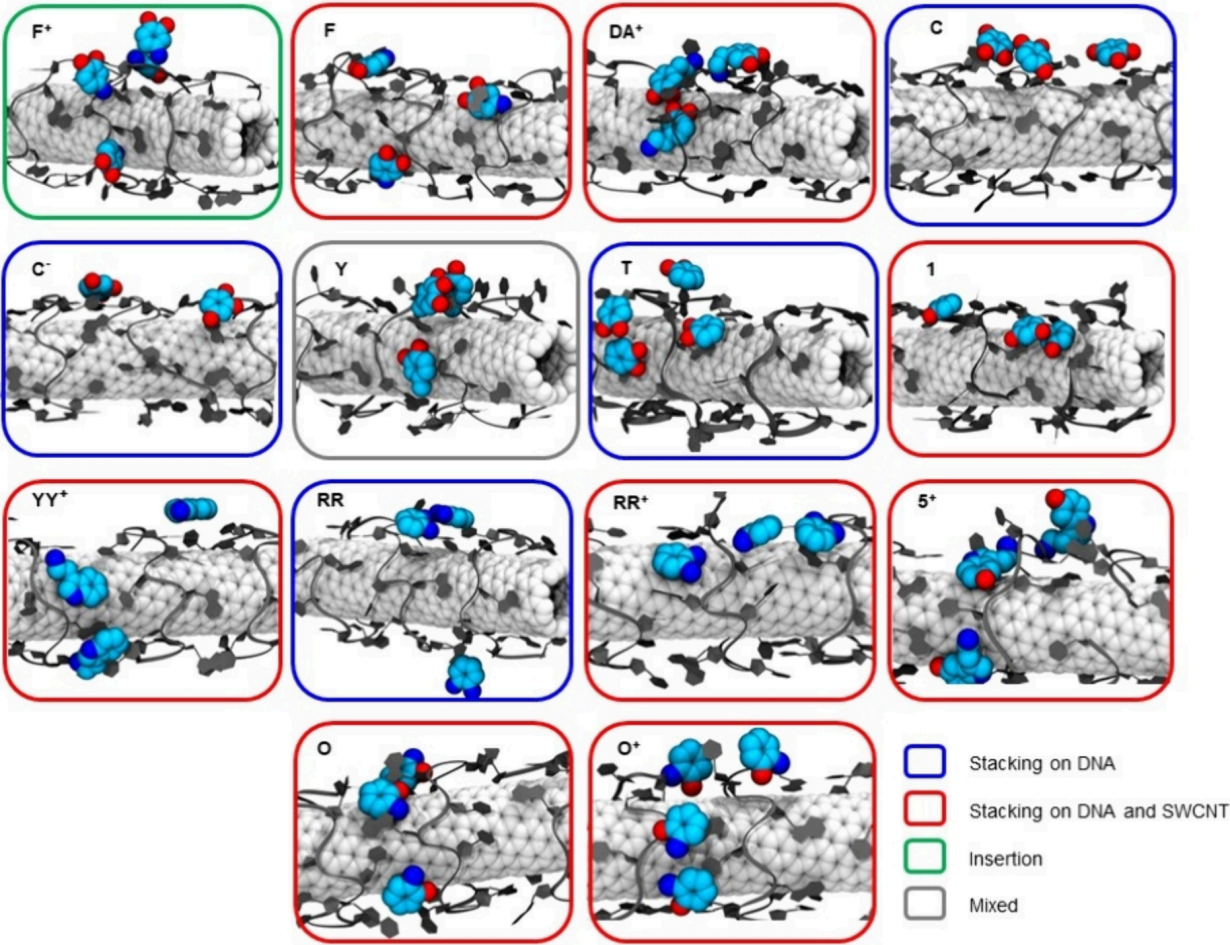
Predominant binding modes for 14 selected analytes with the ssDNA–SWCNT conjugate and the SWCNT surface observed in MD simulations. The SWCNT carbon atoms are shown as white spheres; the (GT)_6_ ssDNA strands are shown as dark gray ribbons. Analyte heavy atoms are shown as van der Waals spheres (C: cyan; N: blue; O: red). The hydrogen atoms are not shown for clarity. Snapshot frame colors correspond to the predominant binding mode observed for the analyte, as defined in the legend. The mixed mode (gray) indicates that there is no predominant binding mode, but the analyte molecules exhibit a mixture of both stacking and insertion interactions.

We began by cataloging all of the predominant binding modes observed when the selected analytes interacted with ssDNA–SWCNT molecular complexes (Figure 4). Figure 4 shows representative snapshots of the preferred binding modes for the 14 analytes we modeled. The observed binding modes fall into two categories: analytes either stacked directly on top of ssDNA nucleotides functionalizing the SWCNT or stacked on the exposed segments of the SWCNT surface. Specifically, molecules **C**, **C**^–^, **T**, and **RR** were primarily observed stacking on the ssDNA nucleotides, while molecules **F**, **DA**^+^, **RR**^+^, **O**, **O**^+^, **5**^+^, **1**, and **YY**^+^ were stacked on both ssDNA and SWCNT surfaces. Interestingly, **F**^+^ favored a distinct insertion mode (Video S1) rather than stacking like **F** (Video S2), although brief instances of sideway stacking on ssDNA bases were occasionally observed (Figure 4). Lastly, analyte **Y** did not exhibit a single predominant binding mode but rather interacted with ssDNA–SWCNT through a combination of binding modes.

It is noteworthy that π-stacking emerged as a prominent binding mode for the ligands studied, largely because a central feature of their molecular structure is an aryl group, which tended to stack on ssDNA bases and the graphitic lattice of SWCNTs. These observations are consistent with our previous computational findings, which demonstrated that dopamine readily stacks on SWCNT and ssDNA–SWCNT surfaces.^12^ In this study, we further observed that the identity and positioning of functional groups around the central aryl motif influenced the preferred binding modes of the analytes. This effect likely stems from the tendency of polar functional groups to engage in directional interactions, shaping the overall orientations of the analytes on nanotube surfaces.

**Figure 5:**
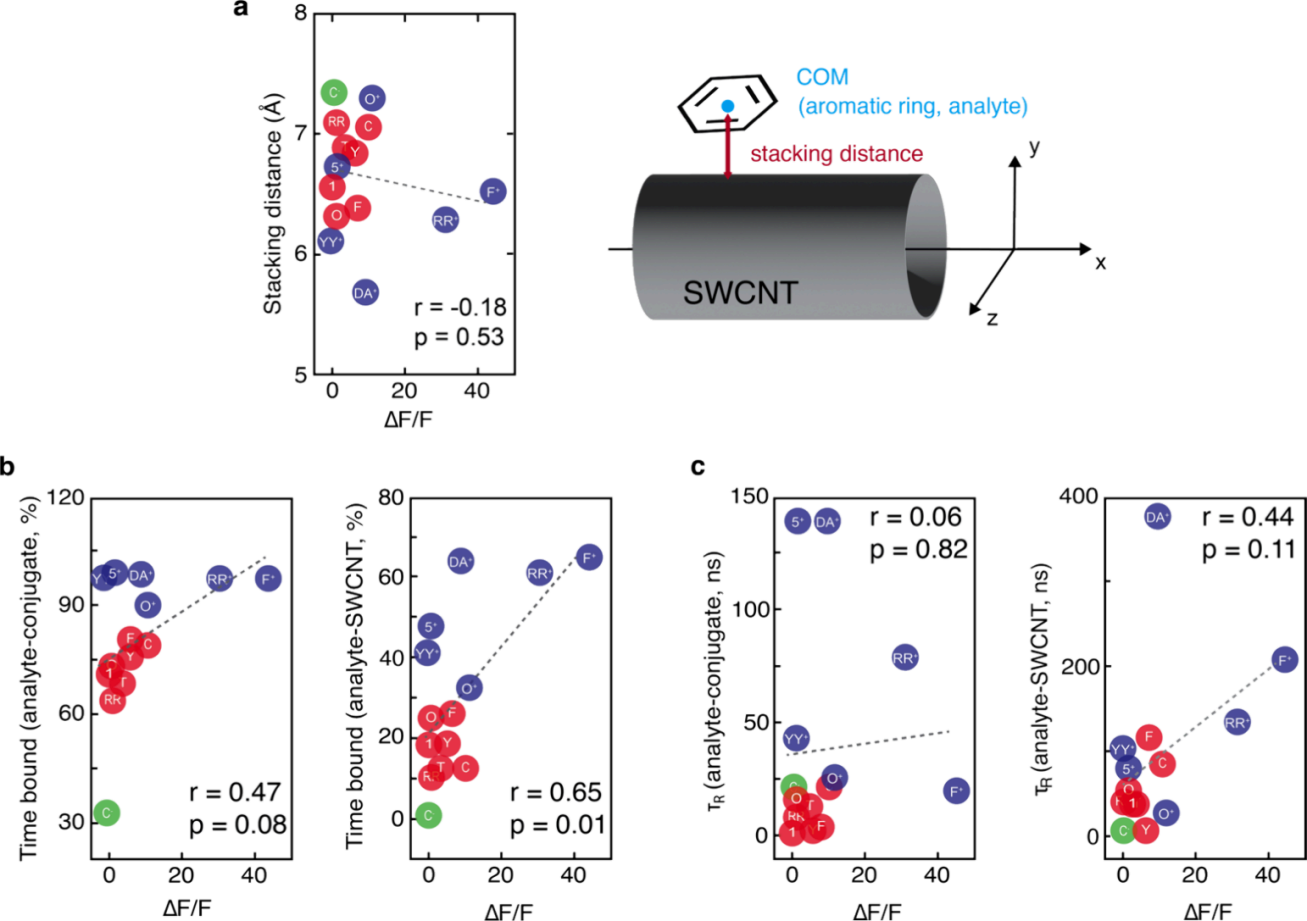
Correlation analysis between MD simulation-derived parameters and experimentally measured optical sensor responses (Δ*F*/*F*) for the (9,4)-SWCNT peak extracted from the full HiPco spectrum. (a) Scatter plot of the average stacking distance of analytes and their Δ*F*/*F* values (mean). The scheme on the right defines the instantaneous stacking distance for the analytes as the shortest distance between the center of mass (COM) of the analyte’s aryl motif and the nanotube surface. The reported distances are averaged only over frames in which the analyte is stacking on the surface within 10 Å and averaged over the six analyte molecules. (b) Scatter plots of the percent of time that analytes are bound to either the ssDNA–SWCNT conjugate (left) or the nanotube surface (right) and Δ*F*/*F* values corresponding to these analytes. (c) Scatter plot of the residence times of analytes when bound to either the ssDNA–SWCNT conjugate (left) or the nanotube surface (right) and Δ*F*/*F* values corresponding to these analytes. In all the plots, scatter points for positively charged, neutral, and negatively charged analytes are shown in blue, red, and green, respectively. The linear regression corresponding to the best fit is shown as a gray dotted line in every plot. The *R*^2^ coefficients, Pearson correlation coefficients (ρ_P_), and *p* values (*p*) are reported in each plot.

To further investigate how molecular structure dictates analyte binding modes, we focused on two analytes: **T** (catechol, *ortho*-hydroxy groups) and **1** (resorcinol, *meta*-hydroxy groups). Experimentally, we found that **1** elicits no optical response, whereas **T** elicits a modest optical response that is smaller than that of **DA**^+^ (−0.056 ± 0.025 vs 0.208 ± 0.032 normalized Δ*F*/*F*, mean ± SD). Visual inspection of the MD trajectories showed distinct binding preferences: **T** predominantly stacked on ssDNA, and **1** exhibited an affinity for both DNA and the SWCNT (Figure 4). To better understand the source of these binding differences, we examined the interactions of **T** and **1** with the sugar–phosphate backbone of ssDNA by calculating the radial distribution functions (*g*(*r*)) for analyte hydroxy groups relative to polar atoms on the ssDNA backbone (O3′, O4′, O5′, P, O1P, and O2P, Figure S16). These *g*(*r*) analyses indicated that hydroxy groups of both **T** and **1** interacted similarly with ssDNA phosphate groups (Figure S16d,g,h). However, the hydroxy groups of **1** showed a higher probability of proximity to (and interaction with) the O3′ and O4′ atoms of ssDNA than those of **T** (Figure S16e,f). All other hydrogen bond interactions in the *g*(*r*) plots were similar for **T** and **1**. Additional quantitative analyses revealed that **1** has a higher average contact area with the SWCNT surface than **T** (Figure S16b) and forms hydrogen-bonding interactions more frequently (Figure S16c), and distance measurements showed that the aryl ring center of mass of **1** is approximately 0.3 Å closer to the SWCNT surface than that of **T**, a finding consistently reproduced across separate 6 μs MD simulations (Figure 5a). The foregoing analysis shows that subtle differences in molecular structure can substantially impact ligand–sensor interactions. Interestingly, although **1** displays modestly better binding interactions with the sensor complex, it fails to produce a better optical response, suggesting that analyte binding alone is insufficient to induce optical perturbations in ssDNA–nanotube biosensors—a key insight in this study.

Analytes with positively charged amine groups (e.g., **F**^+^, **DA**^+^, **YY**^+^, **RR**^+^, **O**^+^, and **5**^+^) interacted with the phosphate backbone of ssDNA through Coulombic attraction (Videos S1 and S3 and Figures S17 and S18). Among these, dopamine (**DA**^+^) demonstrated the strongest binding propensity, primarily driven by directional interactions between the positively charged amine groups and the negatively charged ssDNA phosphate backbone. To investigate the role of these interactions in analyte binding, we analyzed the simulation results for **DA**^+^ and **F**^+^. Both molecules contain positively charged amine groups, yet their binding behaviors differed significantly: **DA**^+^ exhibited stable π-stacking on the nanotube surface (Video S3 and Figure S17b), while **F**^+^ adopted a distinct insertion mode that is not commonly observed in other analytes (Video S1 and Figure S17a). To uncover the basis for these differences, we examined trajectory frames where **DA**^+^ or **F**^+^ engaged in stacking or insertion modes, calculating radial distribution functions to assess the probability of interaction between amine groups and ssDNA phosphate ion (−PO_4_^–^) groups. Our analysis revealed that amine groups in **DA**^+^ predominantly interact with ssDNA phosphate groups during π-stacking on the SWCNT (Figure S18b). In contrast, **F**^+^ displayed such interactions only during insertion into the DNA corona (Figure S18a). This divergence stems from structural differences: the amine group in **DA**^+^ is tethered to the aryl motif via a flexible alkyl chain, whereas in **F**^+^, it is rigid and directly bonded to the aryl ring. These findings again highlight the critical role that electrostatic interactions and molecular structure play in determining the binding modes of analytes within the sensor complex. These modelings suggest that in positively charged analytes, pH-dependent protonation of amine groups is expected to modulate binding behavior. For instance, the amine group in **F**^+^ has a p*K*_A_ of 4.96, while in **DA**^+^, it is 9.31 (Figure 3). This disparity explains the sharp pH sensitivity observed in the optical response of **F**^+^ but not that of **DA**^+^. When **F**^+^ loses its positive charge under elevated pH conditions, its binding affinity to the sensor is dramatically reduced (Video S2). Collectively, these results highlight the interplay among analyte structure, charge state, and environmental factors in governing sensor–analyte interactions.

The neutral molecules (**F**, **T**, **Y**, **1**, **RR**, **C**, and **O**) and the negatively charged molecule (**C**^–^) exhibit a transient binding behavior, and we did not identify a predominant binding interaction that governs their binding behavior. A comparison of the binding of **DA**^+^ and **T** to the SWCNT surface over time is shown in Figure S17k and highlights the notable difference in binding stability between charged and neutral analytes. Despite the transient nature of the interactions for neutral molecules, hydrogen bonds between the hydroxy or amine functional groups and the oxygen atoms of the ssDNA sugar–phosphate backbone or the nitrogen atoms of the ssDNA bases are frequently observed (Figure S16 for **T** and **1** and Figure S17c for **C**). In summary, based on a visual inspection and quantitative analysis of the simulation trajectories, we show that the functional groups present in all ligands dictate their binding mode to the sensor complex.

From the extended MD simulations, we aimed to extract quantitative parameters that could be correlated with experimentally measured optical responses, thereby establishing a nexus between the experiment and simulation that could offer mechanistic insight. Several parameters were derived from the MD simulations for this purpose, including (1) the average distance of each analyte’s aryl ring from the SWCNT surface (Figure 5a), (2) the percentage of time that each analyte spent binding to the ssDNA–SWCNT conjugate or directly to SWCNT surface during the simulation period, and (3) the residence times of each analyte’s interaction with either the SWCNT surface or the entire ssDNA–SWCNT conjugate. Our goal was to determine which of these simulation parameters, in combination with the experimental correlates we identified, could collectively define a “perturbation cross section” for each analyte, defined as the ability of each analyte to perturb the local chemical environment of the nanotube and elicit an optical response.

We first examined the correlation between stacking distance and experimentally measured Δ*F*/*F* values, hypothesizing that the binding proximity to the nanotube surface would be positively correlated with optical perturbations. However, our analysis showed a weak and statistically insignificant negative correlation between the stacking distance and Δ*F*/*F* (Pearson correlation coefficient, *r* = −0.18, Figure 5a). Despite the lack of correlation, a grouped analysis between the positively charged molecules (**F**^+^, **DA**^+^, **YY**^+^, **RR**^+^, **5**^+^, and **O**^+^) and the neutral analytes (**F**, **C**, **Y**, **T**, **1**, **RR**, and **O**) showed that the positively charged molecules bind significantly closer to the SWCNT surface (*p* value = 0.02 from an unpaired *t* test). This finding shows that the proximity of ligand binding to the nanotube surface is not sufficiently predictive of optical response—an important insight from this study.

Further analysis revealed a weak positive correlation between the total percentage of time that analytes were bound to the ssDNA–SWCNT conjugate during 6 μs simulation trajectories and Δ*F*/*F* values (Pearson correlation coefficient, *r* = 0.47, Figure 5b). While not statistically significant, this result may suggest that increased binding duration enhances analyte–sensor interactions, leading to more persistent perturbations in the nanotube environment. Notably, a stronger and statistically significant positive correlation (Pearson correlation coefficient, *r* = 0.65, *p* = 0.01) was observed between the percentage of time that analytes remained bound to the SWCNT surface and Δ*F* and *F* values (Figure 5b). Positively charged analytes displayed significantly longer binding durations with the SWCNT surface than neutral or negatively charged molecules (*p* = 0.002 from an unpaired *t* test), again likely due to favorable interactions between charged amine groups and the ssDNA phosphate backbone.

To further explore ligand binding dynamics, we analyzed binding residence times—defined as the average duration for which an analyte remains bound in a given pose—to distinguish between stable binding interactions and transient, frequent binding events. Correlation analysis between residence times at the ssDNA–SWCNT conjugate and Δ*F*/*F* values showed a negligible relationship (Pearson correlation coefficient, *r* = 0.06, with a nonsignificant *p* value) (Figure 5c). A modestly stronger but still statistically insignificant positive correlation was observed for residence times at the SWCNT surface (Pearson correlation coefficients, *r* = 0.44, Figure 5c, right panel). Among the positively charged analytes, **F**^+^, **DA**^+^, and **RR**^+^ exhibited a significantly longer residence time than the neutral molecules (**F**, **C**, **Y**, **T**, **1**, **RR**, and **O**) (*p* value = 0.001, on an unpaired *t* test). Interestingly, **YY**^+^, **5**^+^, and **O**^+^ had residence times similar to those of neutral molecules, suggesting that the molecules preferentially associated with the ssDNA–SWCNT conjugate rather than the SWCNT surface for majority of the simulation. However, unlike **F**^+^, **DA**^+^_**,**_ and **RR**^+^, their binding interactions were less stable, which was reflected in shorter residence times (Figure 5c). It is notable that **DA**^+^ and **F**^+^ both have two hydroxy groups on adjacent carbons of their aryl rings, whereas **YY**^+^ lacks any substituents, and **5**^+^ and **O**^+^ each contain a single hydroxy group. These structural differences between analytes and their correlated differences in residence times on the SWCNT surface indicate that hydroxy groups play an important role in stabilizing analytes’ binding dynamics to the sensor complex (Figure S17d–j). Indeed, as noted in our analysis of the binding dynamics of **T** and **1**, hydroxy side groups participate in hydrogen bonding with polar atoms of ssDNA (Figure S16), which, working in concert with Coulombic interactions between the analyte and ssDNA, contributes to the overall ligand π-stacking stability (Figure S17d–k).

Considering the important role that polar functional groups play in influencing the nanotube’s immediate chemical environment, we assessed the proximity of polar functional groups to the nanotube surface. Previous studies have demonstrated that the dielectric constant of the nanotube’s environment significantly impacts SWCNT photophysics. For example, Silvera-Batista et al. showed that increasing the dielectric constant of the SWCNT environment from 2 to 5 by changing solvents could reduce the photoluminescence intensity by more than 50%.^44^ This suggests that the polar substituent’s proximity to the SWCNT surface may be an important MD correlate to investigate.

**Figure 6:**
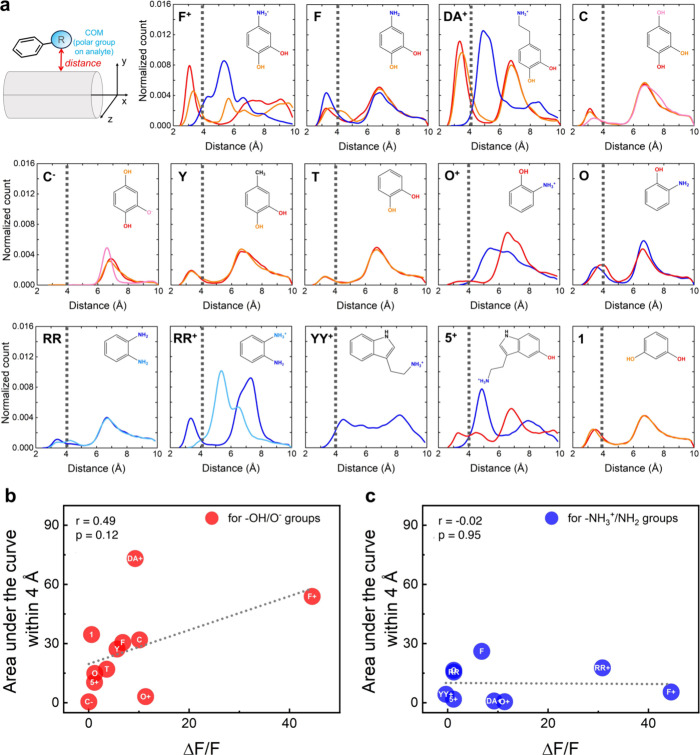
Probability distribution of analyte polar group distances from nanotube surfaces. (a) Distance was defined as the shortest distance between the COM of each analyte’s polar group and the nanotube surface, shown schematically in the top left panel. The distributions consider all six simulated molecules of the analyte and only those frames where the distances are within 10.0 Å. Traces representing amine/ammonium groups are colored blue, and the lines representing hydroxy groups are shown in shades of red. The dotted line at 4.0 Å marks a threshold distance indicating a direct close interaction between the polar group and the nanotube. (b, c) Scatter plots of the areas under the curve within 4 Å of the distance distribution plots shown in panel (a) and their Δ*F*/*F* values (mean, for (9,4)-SWCNT extracted from the full HiPco spectrum) for neutral/negatively charged hydroxy/alkoxide functional groups (red points) and the neutral/positively charged amine/ammonium functional groups (blue). The areas under the curve represent a sum when multiple −OH groups are present in the molecule and when they are both present near the SWCNT surface.

To build on these findings, we measured the distances between the center of mass (COM) of each analyte’s polar functional groups and the nanotube surface in all MD simulations, focusing on trajectory frames in which the groups were found within 10.0 Å of the nanotube surface. The observed distances were then used to generate distributions of the distances between the analyte polar groups and SWCNT surfaces (Figure 6). For instance, molecule **F**^+^ has two polar functional groups: a hydroxy and a charged amine group. An examination of its distribution shows that hydroxy groups have a high probability of being within 3.0–4.0 Å of the SWCNT surface, whereas the amine has a peak at ∼5.2 Å away from the nanotube surface. In contrast, neutral molecule **F** showed similar distance distributions for its hydroxy groups, but the amine group was closer to the nanotube surface, with a maximum at 3.5 Å. These data are consistent with the analyte binding modes discussed earlier, where **F**^+^ molecules exhibit an insertion mode of binding, with the charged amine group projecting away from the nanotube surface. On the other hand, **F** molecules tended to stack on the ssDNA corona or the nanotube surface, with both the hydroxy and amine groups at similar distances from the surface. **DA**^+^ molecules were observed to lie flat on the nanotube surface, but the charged amine group, located two carbon centers away from the aryl ring, projected upward and away from the nanotube surface, interacting with the negatively charged ssDNA. This binding observation is consistent with the polar group distribution profiles (Figure 6). Importantly, experimental data alone could not fully explain why **F** elicits little optical response, whereas **F**^+^ produces a robust response. **F** satisfied most of the heuristics we initially observed in responsive analytes: it features a conjugated aryl motif, *ortho-*hydroxy groups, and a strongly electron-donating substituent (−NH_2_) in conjugation with the aryl motif, rendering the molecule electron rich. Based on these characteristics, **F** would have been expected to elicit a response, yet it did not (Figure 3a). These simulation results now provide critical insights into this discrepancy, revealing that **F** interacts poorly with the sensor complex (Figure 6a). Specifically, contact between the SWCNT surface and the hydroxy substituents of **F** is significantly reduced compared to that of **F**^+^ (Figure 6a,b). Conversely, contact between amino groups and the nanotube surface was less significantly correlated with the optical response, showing that amine/ammonium groups primarily engage DNA phosphate groups (Figure 6c).

**RR**^+^ has two polar groups: an amine and an ammonium. The neutral amine shows a moderate probability of being near the nanotube surface (maximum of 3.4 Å) and a higher probability of being farther away (maximum of 6.4 Å). In contrast, the charged amine has one large peak at 5.4 Å. For molecule **RR** with two neutral amine groups, the distribution is similar for both, with a maximum at 6.4 Å. For **O**^+^, the charged amine is closer to the nanotube (maximum at 5.4 Å) than the neutral hydroxy group (maximum at 6.6 Å). In the neutral molecule **O**, the two polar groups have a similar distribution. For **C** and **C**^–^, deprotonation of one of the *ortho*-hydroxy groups in **C** results in a drastic rearrangement in distribution, with all polar groups in **C**^–^ more than 6.0 Å away from the nanotube surface, suggesting a repulsive and unfavorable interaction. Molecules **Y**, **T**, and **1** all exhibit a similar distribution pattern, with moderate probabilities at ∼3.5 Å from the nanotube surface and much higher probabilities farther away (∼6.5 Å). Comparing the profiles of **DA**^+^ and **Y** and **F** and **F**^+^ further underscores how Coulombic interactions stabilize π-stacking and draw polar functional groups closer to the nanotube surface. Between the isomers **T** and **1**, the hydroxy groups in **1** have a slightly higher probability of being closer to the nanotube surface than those in **T**. For **YY**^+^ molecules, the positively charged amine group is situated two carbon centers from the indolamine. Consequently, stacking on the nanotube surface leads to the polar group being projected away from the nanotube surface, similar to the behavior of **DA**^+^ molecules. A similar observation is noted for the charged amine in compound **5**^+^. These findings further solidify how various binding forces and molecular structure work in tandem to govern how ligands interact with the nanotube surface.

## Discussion

Optical biosensors facilitate advances in various disciplines of biological research by enabling the exploration of questions that are difficult to address with other methods. While many optical biosensors are based on genetically engineered proteins, synthetic optical sensors have also made important contributions. Among these synthetic biosensors, SWCNTs possess useful photophysical attributes that make them particularly well suited for applications in biology.^1^ Consequently, SWCNT-based biosensors have been developed for a wide range of bioanalytes, including reactive oxygen species,^10,45^ small biomolecules and lipids,^11,13,16,46^ neuropeptides,^14^ proteins,^47−49^ disease biomarkers,^50,51^ and even bacteria and viruses.^52,53^

Despite the growing list of analytes for which SWCNT-based sensors have been developed, the mechanisms behind their molecular recognition and optical modulation are poorly understood. In this work, we studied (GT)_6_–SWCNT bio–nano hybrids, which exhibit vigorous fluorescence modulation when exposed to catecholamines, a family of biologically important small molecules. Our goal was to enhance the understanding of sensor optical modulation by systematically exploring the relationship between ligand properties and optical responses. We complemented our experimental findings with MD simulations to rationalize our observations and gain valuable insights that lead to a mechanistic understanding of sensor function.

Our experiments demonstrated that the electron densities of the aryl rings in catechols positively correlated with the amplitude of their optical response. Electron-deficient catechols elicited lower optical responses compared to electron-rich ones (Figure 2 and Figure S7). Given the strong correlation between catechol electron densities and their reduction potential, we investigated whether the oxidation of catechols and subsequent electron transfer to nanotubes might underlie the observed optical modulation. However, we found that catechols do not undergo oxidation upon exposure to excited ssDNA–SWCNT conjugates. Previous studies have shown that some small-molecule reducing agents increase the brightness of nanotube suspensions.^54,55^ However, whether redox reactions occur between these analytes and the ssDNA–SWCNT complex has remained unclear. Our finding using catechol-bearing substrates suggests that the optical sensitivity of ssDNA–SWCNT conjugates to redox-active small molecules is likely driven by a transient perturbative process rather than being caused by electron transfers that characterize true redox reactions. Moreover, through a systematic exploration of derivatized catechols and related compounds, we defined molecular structure and charge characteristics that play key roles in ligand–sensor interactions and identified aminocatechols and phenylenediamines as substrates that can elicit robust optical responses from ssDNA–SWCNT conjugates, thereby expanding the substrate scope detectable by (GT)_*N*_–SWCNT bio–nano conjugates. While these may lead to new sensor applications for analytes that contain these motifs, they also highlight potentially cross-reactive compounds that may interfere with sensor applications, especially in chemically diverse biological applications.

Optical biosensors fall into two major groups: those based on molecular recognition and those based on chemical reactivity, known as activity-based sensing.^56^ While traditional optical biosensors typically rely on a lock-and-key-type molecular recognition process, activity-based sensors detect molecular reactivity between the sensor and the analyte.^56,57^ Regarding ssDNA–SWCNT catecholamine sensors, our data indicated a strong correlation between catechol redox activity and optical response, suggesting an activity-based model may be a fit. However, the absence of detectable oxidized catechol products was not fully consistent with this model. Moreover, we observed that the optical response of certain catechols was highly sensitive to solution pH, indicating that the protonation state (charge) of the various substituents on the aryl group plays a key role in the molecular recognition and optical modulation process.

To gain a better understanding and develop an integrated model of sensor function, we employed MD simulations. Our goal was to first characterize stable binding modes between a carefully selected group of analytes and ssDNA–SWCNT conjugates. We then sought to identify the analyte–sensor interaction parameters that correlate with experimentally measured optical responses. From binding interactions, we identified two primary modes of association between analytes and ssDNA–SWCNT conjugates: stacking on ssDNA bases and stacking on nanotube surfaces. In both cases, we found that the molecular charge and the polarity of substituents strongly influenced the stacking stability of the molecules. This explained the sensor’s pH dependence that we observed experimentally. Specifically, positively charged substituents (ammonium groups) strongly interact with ssDNA phosphate groups, affecting both the stability of binding and binding residence times. Moreover, transient hydrogen bonds between analytes and the sensor complex have emerged as a key stabilizing force in ligand–sensor interactions.

Notably, some analytes with positively charged amine groups could exhibit relatively stable binding through interactions with ssDNA phosphate groups but elicited small (e.g., **5**^+^) or no (e.g., **YY**^+^) optical response. Conversely, some analytes without charged amine groups (e.g., **C**) were able to generate a strong optical response. This indicated that the perturbation cross section of an analyte is not a simple function of its ability to bind to ssDNA–SWCNT conjugates through electrostatic and hydrogen bond interactions. Instead, the characteristics of substituents on the aryl group play an important role, consistent with experimental observation that substituents significantly influence optical response. Further analysis revealed that the proximity of polar substituents to nanotube surfaces correlated positively with the optical response. This indicates that molecules that have a high perturbation cross section (e.g., **DA**^+^ and **F**^+^) not only stably bind ssDNA–SWCNT conjugates but also exhibit a higher density of polar substituents close to the nanotube surface (Figure 6).

## Conclusions

In summary, analytes with a high perturbation cross section that strongly modulates SWCNT optical responses exhibit distinct structural and electronic features. These include vicinal hydrogen bond donors positioned on a π-conjugated scaffold. Electron-donating substituents, particularly in *para*-positions, enhance responses by increasing electron density and raising HOMO energy levels. Sterically bulky analytes are well tolerated, indicating an accessible binding pocket, while nonconjugated systems or hydrogen bond acceptors weaken responses. Although optical responses strongly correlate with the reduction potential and electrochemical properties, the analytes themselves are not oxidized during modulation. These attributes highlight the importance of hydrogen bonding, conjugation, and electronic properties in enabling effective SWCNT optical modulation. To synthesize all experimental observations into a mechanistic insight, we used MD simulations. These simulations revealed that stable ligand binding, which primarily relies on Coulombic interactions, hydrogen bonding, and π-stacking, is necessary but not sufficient to elicit a response. The effectiveness of a ligand in eliciting an optical response depends on its substituent’s electronic properties and proximity to the nanotube surface in its bound state. This interplay of binding and electronic effects underpins the unique optical modulation mechanism of ssDNA–SWCNT sensors.

Several explanations could account for the observed dependence of the sensor response on the electron-donating or electron-withdrawing character of the substituents. One possibility is that electron-donating substituents enhance the π-stacking stability of the aryl ring on the nanotube surface, consistent with well-known substituent effects on π-interaction between aromatic rings.^58^ This stable stacking may more efficiently displace water molecules from the nanotube surface, transiently reducing the surface dielectric constant and thereby increasing the optical output. This hypothesis is supported by previous findings, where we showed that dopamine binding outcompetes sodium cholate binding to nanotube surfaces in ssDNA–SWCNT conjugates.^12^ Another potential explanation is that stably bound and electron-rich aryl motifs could coordinate with deleterious surface defects on SWCNTs, locally and transiently altering the nanotube bandgap, which could increase optical output. Alternatively, electron-rich substituents themselves, instead of the π-stacked aryl groups, may be responsible for displacing water or transiently mitigating the effect of surface defects, thus enhancing the nanotubes’ brightness. These potential mechanisms highlight the complexity of interaction between analytes and ssDNA-conjugated nanotubes, suggesting that multiple factors may contribute to the sensor response. In conclusion, our findings indicate that optical responses in ssDNA–SWCNT conjugates depend on both molecular binding events, similar to traditional optical sensors, and the chemical properties (structure, charge, and electron density) of the analyte, similar to activity-based sensing models.

These findings suggest promising avenues to enhance the performance of existing sensors and guide the development of new sensors. Two key opportunities emerged from our results. First, in sensor development, much of the effort typically focuses on identifying molecular recognition motifs for the target analyte with the assumption that binding of the ligand to these motifs is expected to drive the optical signal in the conjugated emitter. While this strategy has proven effective for the development of genetically encoded probes, it has been less successful in the design of SWCNT-based probes. Our study reveals that ligand binding is only part of the equation in designing SWCNT-based optical probes. This motivates the possibility of incorporating electrochemically active motifs into sensor design to complement the perturbative effect of ligand binding. Specifically, our findings highlight the unique efficacy of catechol-like, electron-rich ligands to elicit a nanotube optical response. This suggests that incorporating them as linkers between an analyte sensing motif and the nanotube may serve as a universal strategy to amplify optical modulation upon ligand binding. This approach draws inspiration from linker optimization strategies commonly used in genetically encoded biosensors, offering a framework to exploit catechol-like motifs as general linkers in nanotube-based biosensors. Second, catechol-bearing motifs can be covalently attached to analytes for which a biosensor is sought, enabling the adaptation of ssDNA–SWCNT catechol sensors as versatile generic sensors for a broader class of molecules. This adaptability expands the range of detectable substrates, broadening the application scope of this class of sensors with minimal adjustments.

## Methods

### Experimental Materials and Methods

#### (GT)_6_–SWCNT Sensor Preparation

All measurements reported throughout this study used HiPco SWCNTs except where noted in the chiral-enriched experiments (Figure S14). HiPco raw SWCNTs (NanoIntegris) were hydrated with H_2_O (Milli-Q, 18.2 Ω, 1 g/50 mL) and stored sealed at room temperature until use. Desalted (GT)_6_ ssDNA (IDT) was dissolved in 0.1 M NaCl (1 mg/60 μL) and frozen at −20 °C until use. Hydrated SWCNTs (4–5 mg) were combined with (GT)_6_ (1 mg) and 0.1 M NaCl (1 mL/mg ssDNA) in a 12 × 75 × 1 mm glass culture tube and bath sonicated (Branson 1800) for 20 min on high at room temperature. Contents were then transferred to a microwave tube (Biotage conical 0.5–2.0 mL, Part No. 352016) and probe sonicated (Sonics Vibracell VCX 230, 3 mm probe, 50% amplitude, centered and tip at 15 mm from the bottom of tube) for 15 min in an ice bath. Afterward, the contents were transferred to a 1.5 mL microcentrifuge tube and centrifuged in a fixed-angle rotor at 20,000 rcf, for 1 h, at 4 °C. The supernatant was transferred to a new 1.5 mL centrifuge tube, and the pellet was discarded. The suspension was then recentrifuged for an additional hour at 20,000 rcf, 4 °C, on the same rotor. The supernatant was then removed, and the second pellet was discarded. To account for possible differences in preparation, multiple 1 mL preparations were prepared and combined to create one bulk solution (10 preparations total). An aliquot of this bulk supernatant was diluted (10×), and the absorbance measured at 632 nm (NanoDrop One C) was used to estimate stock concentrations. The bulk supernatant solution was diluted to 100 ppm with 0.1 M NaCl and stored at 2–8 °C until use.

#### Analyte Stock Preparation

All analytes were made from commercially available vendors and used without further purification. See Table S4 for a list of the compounds, sources, and calculated values. All analytes were freshly made into 10 mM stocks in dimethyl sulfoxide (DMSO, spectrophotometric grade), aliquoted into argon-filled amber 1.5 mL tubes, topped with argon again, and frozen at −20 °C for up to 3 months. For use in assay, samples were thawed and diluted to 1 mM working concentrations with DMSO, which were then added to respective wells of a 96-well plate for a final 10 μM concentration of the analyte in a 99% 0.1 M NaCl:1% DMSO matrix. Final concentrations are nominal, and we did not account for potential differences in analyte solubility.

#### pH Measurements

All pH measurements were taken with an Orion Star A111 pH meter by using an Orion PerpHecT ROSS combination pH microelectrode capable of measurements in a 96-well plate. All measurements were taken after the probe had been freshly calibrated using 4.01, 7.00, and 10.01 standards. pH measurements were taken of pH-adjusted 10 ppm (GT)_6_–SWCNT prior to aliquoting into plate wells. This measurement had to remain unchanged (±0.05 pH units) for 1 h to ensure that equilibrium had been reached prior to aliquoting. After substrates were added and all measurements taken, the pH of individual wells was then measured. The average values of the three wells used for each analyte were used as the average pH and response for the respective compound. The measurements were typically taken between 65 and 90 min after the analyte had been initially added to the 10 ppm solution.

#### Plate Reader Solution-Phase Fluorescence Measurements

All readings were taken on a custom-built near-infrared 96-well plate reader. All readings were taken with 10 ppm (GT)_6_–SWCNT in 0.1 M NaCl (198 μL), *n* = 3, the readings taken with a 658 nm laser, 52.4 mW, 1000 ms exposure, 3 averages per read. Additives were added from DMSO stock (2 μL of 1 mM) unless otherwise noted. A well containing 0.1 M NaCl (198 μL) and DMSO (2 μL) were used for blank subtraction. Baseline measurements were taken approximately 15 min after the 10 ppm solution was aliquoted (*F*_0_). Analytes were then added to all wells, and measurements (*F*) were taken at 4, 8, 15, 30, 45, and 60 min. This study used the 30 min read values as *F*, and the same trends were seen at other time points. Δ*F*/*F* values were computed as (*F* – *F*_0_)/*F*_0_ from integrated spectra. In MD simulations, the (9,4)-SWCNT species was modeled. Therefore, Δ*F*/*F* values were recomputed for the (9,4) peak and were used in the results of Figure 5 and related Supporting Information (SI) figures. Each 96-well plate contained dopamine (**DA**) for normalization, pyrogallol (**MM**) as a positive control, and octopamine (**OO**) as a negative control.

#### SWCNT pH Stability Measurements

All pH measurements were taken with an A111 pH meter using an Orion PerpHecT ROSS combination pH microelectrode. All measurements were taken after the probe had been freshly calibrated using 4.01, 7.00, and 10.01 standards. pH measurements were taken of 1 mL aliquots of pH-adjusted 10 ppm (GT)_6_–SWCNT in 0.1 M NaCl after adjustment with HCl or NaOH. This measurement had to remain unchanged (±0.05 pH units) for 1 h to ensure that equilibrium had been reached. Afterward, the samples were incubated at room temperature at their respective pH for 1 h, followed by centrifugation at 20,000 rcf, 60 min, 16 °C. The supernatant optical density was then remeasured using absorbance at 632 nm (NanoDrop One C) to calculate concentrations. pH was then remeasured to ensure that no drift had occurred.

#### ELISA

The dopamine ELISA kit (ImmuSmol SAS, Bordeaux, France) was run experimentally and processed in accordance with the manufacturer’s guidelines and standard operating procedure. For sample preparation, a 96-well plate was prepared with wells containing 198 μL of 10 ppm (GT)_6_–SWCNT in 0.1 M NaCl that were spiked with **DA** (10 μM final concentration) or DQ (10 μM final concentration) as a substrate in DMSO (2 μL of 1 mM) and 198 μL of 0.1 M NaCl containing **DA** (10 μM final concentration) as a substrate in DMSO (2 μL of 1 mM). For each respective substrate run, one well was exposed to 104.8 mW of 658 nm laser for 1 h and the other was exposed no laser, with mixing via pipet aspiration every 15 min. After 1 h, the contents of each well were filtered through a 100 kDa molecular weight cutoff (MWCO) centrifuge cartridge to remove SWCNTs, and the filtrate was collected. 28.2 μL of the filtrate was combined with 235 μL of ethylenediaminetetraacetic acid (EDTA, 10 mM), 235 μL of sodium metabisulfite (40 mM), and 1851.8 μL of water, before being frozen at −80 °C. Dilution was necessary for the sample to fit within the kit’s dynamic range, and EDTA and sodium metabisulfite were added to prevent dopamine degradation as per the manufacturer’s protocol. Samples were then thawed to room temperature and run in a quantitative DA ELISA kit following the manufacturer’s protocol. Standards and samples were run as *n* = 3 and quality control (QC) samples as *n* = 2. The ELISA plates were read using a Tecan Spark microplate reader at 450 nm with a 625 nm reference. Data were then processed using Graphpad Prism 10. Standards were treated with four-parameter logistic regression as per the manufacturer’s protocol, and the QC, exposed, and unexposed samples were interpolated from this curve.

#### p*K*_A_ Modeling

p*K*_A_ modeling and chemoinformatic values were generated using the Chemicalize software from Chemaxon. Values were computed between March 2023 and April 2024.^43^

#### HPLC

Aqueous solutions of **DA** (40 mM) and NaIO_4_ (35 mM) were prepared. A 500 μL aliquot of the **DA** solution was combined with a 500 μL aliquot of NaIO_4_, and the solution was vortexed and kept at room temperature for 10 min before being taken for HPLC analysis. A 500 μL aliquot of **DA** (40 mM) was diluted with 500 μL of H_2_O to produce a 20 mM solution of **DA**. Two wells on opposite sides of a 96-well plate (e.g., C2 and C11) were loaded with 10 ppm of (GT)_6_–SWCNT in H_2_O (198 μL) and 2 μL of the 20 mM **DA** solution was added to each. One well was exposed to a 658 nm laser (104.8 mW), and one was not. The wells were agitated with a pipet every 15 min for 1 h total. Afterward, the contents of the wells were filtered through 100 kDa MWCO centrifuge filters (15,000 rcf, 4 °C, 2 min, fixed-angle rotor) to remove the SWCNTs and the filtrate was taken for analysis by HPLC. Please refer to the SI for HPLC runs and conditions.

### Computational Materials and Methods

#### Atomistic Models of (GT)_6_–(9,4)-SWCNT Systems with Analyte Molecules

The initial configuration of a (9,4)-SWCNT wrapped with three (GT)_6_ chains was taken from previously reported results.^12^ The small molecules were built with the GaussView software.^59^ All of the (GT)_6_–SWCNT systems, each prepared with six analyte molecules, were solvated and neutralized in 0.1 M NaCl aqueous solution with the TIP3P water model, using the solvate and ionize VMD plug-ins, respectively. The total number of atoms in each of these systems is listed in Table S5.

#### Classical MD Simulations

Atomistic simulations were performed with each of the prepared systems to gain insight into the molecular-level behavior of the nanosensor conjugate as it binds to the analyte molecules. The systems were described with CHARMM36 force field parameters^60,61^ as they have been successfully used to model interactions between ssDNA molecules and SWCNTs in previous studies.^12,62−65^ The parameters for the analyte compounds were generated from the CGenFF Web site,^66^ based on CHARMM36 general force field parameters. The simulations were performed with the NAMD2.13 package^67^ using Langevin dynamics in the NpT ensemble, where the value of the Langevin constant γ_Lang_ was set at 1.0 ps^–1^, the pressure remained constant at 1 bar, and the temperature remained constant at 298 K. The integration time step was set to 2 fs, and Coulomb and van der Waals nonbonded interactions were evaluated every one- and two-time steps, respectively, for all atoms within a 12 Å cutoff distance. The long-range Coulomb interactions were evaluated using the particle-mesh Ewald (PME) method,^68^ with periodic boundary conditions applied in all directions. After 5000 steps of minimization, solvent molecules were equilibrated for 0.1 ns around the ssDNA–SWCNT conjugate. For this purpose, the atoms were restrained using harmonic forces with a spring constant of 1 kcal (mol·Å)^−1^. Next, the systems were equilibrated in production MD runs, with harmonic wall restraints applied on the ssDNA side chains (A and C) and the small molecules. For the harmonic wall restraints, upper and lower walls were defined at 19 and −19 Å, respectively, and a spring constant of 10 kcal (mol·Å)^−1^ was applied. The lengths of all simulations are listed in Table S5.

#### Contact Area Calculations

Contact areas between two selections of atoms A and B (e.g., analyte molecules, ssDNA nucleotides, and SWCNT surface) at time *t, s*_contact area_(*t*), were calculated for the whole MD trajectories (∼6 μs) based on the following equation:
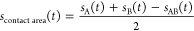
1where *s*_A_(*t*) and *s*_B_(*t*) are the solvent-accessible surface areas (SASA) of atoms within selections A and B at time *t*, respectively. *s*_AB_(*t*) represents the SASA of both selections A and B altogether. The contact areas were calculated with the SASA VMD plug-in, where the van der Waals radius of atoms was defined as 1.4 Å to designate the points on a sphere that are accessible to the solvent.

#### Distance Calculations

To quantify the binding modes visually observed from the MD trajectories and also to analyze the effect of the polar groups, we calculated distances between the COMs of selected parts of the analyte molecules (aryl ring, polar groups) and the SWCNT surface at time *t*:
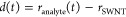
2where *r*_analyte_(*t*) is the radial distance of the COM of the selected analyte atoms at time *t*, defined in the cylindrical coordinate system, and *r*_SWNT_ is the radius of the (9,4)-SWCNT.

#### Calculation of the Percentage of Binding Time for Analytes Binding to the SWCNT Surface or ssDNA–SWCNT Corona

Here, we quantify the percentage of time in a total trajectory for which the analyte molecules are bound to the sensor conjugate. From the contact area calculations, we imposed a condition to exclude the frames where the analyte molecules are not binding to either the ssDNA–SWCNT corona or the SWCNT surface and are located somewhere in the water box. We concurred that if the analyte molecules were far away from the ssDNA–SWCNT conjugate in the water box, the contact area with ssDNA–SWCNT/SWCNT would be near 0, so we excluded all those frames and counted only the number of times that the contact areas were greater than 1, which signified that the analyte molecules were in the proximity of the ssDNA–SWCNT conjugate. Ultimately, we divided the count of times that the analyte molecules were bound to the nanosensor conjugate by the total number of frames to get the percentage of binding time for each molecule of each analyte type.

#### Residence Time Calculations for Binding to the SWCNT Surface or ssDNA–SWCNT Corona

The residence time (τ_R_) for an analyte binding to a specific target is defined as the time it remains in a specific contact position with its target.^69,70^ Mathematically, it is the inverse of the dissociation rate (*k*_off_) of the analyte–target complex. *k*_off_ is the inverse of the average of time the analyte molecules are bound to the target in different binding events (*t*_off_).^71^ So, in turn, residence time becomes equivalent to *t*_off_.
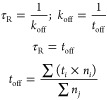
3

In eq 3, *t_i_* is the duration of a binding event of certain duration *i*, and *n_i_* is the total number of binding events with duration *i*. *n_j_* is the number of binding events with different durations (*j* = *i*_1_ + *i*_2_ + *i*_3_ + ⋯).

From the contact area calculations, we extracted the frames for which the analyte molecules are binding to the SWCNT surface or the ssDNA–SWCNT conjugate by imposing certain conditions. To qualify as a binding event, the contact area between the analyte molecules and the ssDNA–SWCNT corona or the SWCNT surface must be greater than 30 Å^2^, and for the analyte molecules with amine groups, the distance between the amine groups and the SWCNT surface must be less than 10.5 Å. The duration of each binding event is then extracted and used to calculate the *t*_off_ or residence time using eq 3. We used our own Python codes for all of these calculations. For each analyte type, all the binding events of all the six molecules were summed up together to calculate *t*_off_.

## Data Availability

The data underlying this study are available in the published article and its Supporting Information. MD simulation videos (Videos S1, S2, and S3) are available on figshare at the following address: https://figshare.com/articles/media/Molecular_Dynamics_Simulation_Videos_for_F_F_and_DA_/28083272.

## Supporting Information Available

Examples of baseline and response fluorescence, dose–response curves, chemoinformatic plots, HPLC conditions and raw data, plots supporting electrochemical correlations, pH–response curves and stability analysis, Chemaxon-derived microspecies distributions, MD simulation results, and tables containing numerical data related to Figures 1 and 2 (PDF)

## Notes

A preliminary draft of this work was previously deposited to the preprint server ChemRxiv.^72^

## References

[ref1] Krasley A. T.; Li E.; Galeana J. M.; Bulumulla C.; Beyene A. G.; Demirer G. S. Carbon Nanomaterial Fluorescent Probes and Their Biological Applications. Chem. Rev. 2024, 124 (6), 3085–3185. 10.1021/acs.chemrev.3c00581. 38478064 PMC10979413

[ref2] Gao Z. Advances in surface-coated single-walled carbon nanotubes as near-infrared photoluminescence emitters for single-particle tracking applications in biological environments. Polym. J. 2018, 50 (8), 589–601. 10.1038/s41428-018-0052-8.

[ref3] Jin H.; Heller D. A.; Strano M. S. Single-Particle Tracking of Endocytosis and Exocytosis of Single-Walled Carbon Nanotubes in NIH-3T3 Cells. Nano Lett. 2008, 8 (6), 1577–1585. 10.1021/nl072969s. 18491944

[ref4] Paviolo C.; Ferreira J. S.; Lee A.; Hunter D.; Calaresu I.; Nandi S.; Groc L.; Cognet L. Near-Infrared Carbon Nanotube Tracking Reveals the Nanoscale Extracellular Space around Synapses. Nano Lett. 2022, 22 (17), 6849–6856. 10.1021/acs.nanolett.1c04259. 36038137 PMC9479209

[ref5] Godin A. G.; Varela J. A.; Gao Z.; Danné N.; Dupuis J. P.; Lounis B.; Groc L.; Cognet L. Single-nanotube tracking reveals the nanoscale organization of the extracellular space in the live brain. Nat. Nanotechnol. 2017, 12 (3), 238–243. 10.1038/nnano.2016.248. 27870840

[ref6] Ackermann J.; Metternich J. T.; Herbertz S.; Kruss S. Biosensing with Fluorescent Carbon Nanotubes. Angew. Chem., Int. Ed. 2022, 61 (18), e202112372 10.1002/anie.202112372. PMC9313876 34978752

[ref7] Kruss S.; Hilmer A. J.; Zhang J.; Reuel N. F.; Mu B.; Strano M. S. Carbon nanotubes as optical biomedical sensors. Adv. Drug Delivery Rev. 2013, 65 (15), 1933–1950. 10.1016/j.addr.2013.07.015. 23906934

[ref8] Zhang J.; Boghossian A. A.; Barone P. W.; Rwei A.; Kim J. H.; Lin D.; Heller D. A.; Hilmer A. J.; Nair N.; Reuel N. F.; Strano M. S. Single molecule detection of nitric oxide enabled by d(AT)15 DNA adsorbed to near infrared fluorescent single-walled carbon nanotubes. J. Am. Chem. Soc. 2011, 133 (3), 567–581. 10.1021/ja1084942. 21142158

[ref9] Kim J.-H.; Heller D. A.; Jin H.; Barone P. W.; Song C.; Zhang J.; Trudel L. J.; Wogan G. N.; Tannenbaum S. R.; Strano M. S. The rational design of nitric oxide selectivity in single-walled carbon nanotube near-infrared fluorescence sensors for biological detection. Nat. Chem. 2009, 1 (6), 473–481. 10.1038/nchem.332. 21378915

[ref10] Jin H.; Heller D. A.; Kalbacova M.; Kim J.-H.; Zhang J.; Boghossian A. A.; Maheshri N.; Strano M. S. Detection of single-molecule H2O2 signalling from epidermal growth factor receptor using fluorescent single-walled carbon nanotubes. Nat. Nanotechnol. 2010, 5 (4), 302–309. 10.1038/nnano.2010.24. 20208549 PMC6438196

[ref11] Kruss S.; Landry M. P.; Vander Ende E.; Lima B. M. A.; Reuel N. F.; Zhang J.; Nelson J.; Mu B.; Hilmer A.; Strano M. Neurotransmitter Detection Using Corona Phase Molecular Recognition on Fluorescent Single-Walled Carbon Nanotube Sensors. J. Am. Chem. Soc. 2014, 136 (2), 713–724. 10.1021/ja410433b. 24354436

[ref12] Beyene A. G.; Alizadehmojarad A. A.; Dorlhiac G.; Goh N.; Streets A. M.; Král P.; Vuković L.; Landry M. P. Ultralarge Modulation of Fluorescence by Neuromodulators in Carbon Nanotubes Functionalized with Self-Assembled Oligonucleotide Rings. Nano Lett. 2018, 18 (11), 6995–7003. 10.1021/acs.nanolett.8b02937. 30350638 PMC6771428

[ref13] Jeong S.; Yang D.; Beyene A. G.; Del Bonis-O’Donnell J. T.; Gest A. M. M.; Navarro N.; Sun X.; Landry M. P. High-throughput evolution of near-infrared serotonin nanosensors. Sci. Adv. 2019, 5 (12), 3771–3789. 10.1126/sciadv.aay3771. PMC6920020 31897432

[ref14] Mun J.; Navarro N.; Jeong S.; Ouassil N.; Leem E.; Beyene A. G.; Landry M. P. Near-infrared nanosensors enable optical imaging of oxytocin with selectivity over vasopressin in acute mouse brain slices. Proc. Natl. Acad. Sci. U. S. A. 2024, 121 (26), e2314795121 10.1101/2022.10.05.511026. 38905241 PMC11214003

[ref15] Iverson N. M.; Barone P. W.; Shandell M.; Trudel L. J.; Sen S.; Sen F.; Ivanov V.; Atolia E.; Farias E.; McNicholas T. P.; Reuel N.; Parry N. M. A.; Wogan G. N.; Strano M. S. In vivo biosensing via tissue-localizable near-infrared-fluorescent single-walled carbon nanotubes. Nature Nanotechnology 2013 8:11 2013, 8 (11), 873–880. 10.1038/nnano.2013.222. PMC4066962 24185942

[ref16] Zhang J.; Landry M. P.; Barone P. W.; Kim J.-H.; Lin S.; Ulissi Z. W.; Lin D.; Mu B.; Boghossian A. A.; Hilmer A. J.; Rwei A.; Hinckley A. C.; Kruss S.; Shandell M. A.; Nair N.; Blake S.; Şen F.; Şen S.; Croy R. G.; Li D.; Yum K.; Ahn J.-H.; Jin H.; Heller D. A.; Essigmann J. M.; Blankschtein D.; Strano M. S. Molecular recognition using corona phase complexes made of synthetic polymers adsorbed on carbon nanotubes. Nat. Nanotechnol. 2013, 8 (12), 959–968. 10.1038/nnano.2013.236. 24270641 PMC5051352

[ref17] Nakatsuka N.; Yang K.-A.; Abendroth J. M.; Cheung K. M.; Xu X.; Yang H.; Zhao C.; Zhu B.; Rim Y. S.; Yang Y.; Weiss P. S.; Stojanović M. N.; Andrews A. M. Aptamer–field-effect transistors overcome Debye length limitations for small-molecule sensing. Science 2018, 362 (6412), 319–324. 10.1126/science.aao6750. 30190311 PMC6663484

[ref18] Zhao M.; Chen Y.; Wang K.; Zhang Z.; Streit J. K.; Fagan J. A.; Tang J.; Zheng M.; Yang C.; Zhu Z.; Sun W. DNA-directed nanofabrication of high-performance carbon nanotube field-effect transistors. Science 2020, 368 (6493), 878–881. 10.1126/science.aaz7435. 32439791

[ref19] Lin Z.; Yang Y.; Jagota A.; Zheng M. Machine Learning-Guided Systematic Search of DNA Sequences for Sorting Carbon Nanotubes. ACS Nano 2022, 16 (3), 4705–4713. 10.1021/acsnano.1c11448. 35213805

[ref20] Yang F.; Wang M.; Zhang D.; Yang J.; Zheng M.; Li Y. Chirality Pure Carbon Nanotubes: Growth, Sorting, and Characterization. Chem. Rev. 2020, 120 (5), 2693–2758. 10.1021/acs.chemrev.9b00835. 32039585

[ref21] Lin Z.; Beltran L. C.; De Los Santos Z. A.; Li Y.; Adel T.; Fagan J. A.; Hight Walker A. R.; Egelman E. H.; Zheng M. DNA-guided lattice remodeling of carbon nanotubes. Science 2022, 377 (6605), 535–539. 10.1126/science.abo4628. 35901135 PMC9872717

[ref22] Gong X.; Renegar N.; Levi R.; Strano M. S. Machine learning for the discovery of molecular recognition based on single-walled carbon nanotube corona-phases. npj Comput. Mater. 2022, 8 (1), 135 10.1038/s41524-022-00795-7.

[ref23] Hendler-Neumark A.; Bisker G. Fluorescent Single-Walled Carbon Nanotubes for Protein Detection. Sensors 2019, 19 (24), 5403 10.3390/s19245403. 31817932 PMC6960995

[ref24] Kelich P.; Jeong S.; Navarro N.; Adams J.; Sun X.; Zhao H.; Landry M. P.; Vuković L. Discovery of DNA–Carbon Nanotube Sensors for Serotonin with Machine Learning and Near-infrared Fluorescence Spectroscopy. ACS Nano 2022, 16 (1), 736–745. 10.1021/acsnano.1c08271. 34928575

[ref25] Kelich P.; Adams J.; Jeong S.; Navarro N.; Landry M. P.; Vuković L. Predicting Serotonin Detection with DNA-Carbon Nanotube Sensors across Multiple Spectral Wavelengths. J. Chem. Inf. Model. 2024, 64 (10), 3992–4001. 10.1021/acs.jcim.4c00021. 38739914

[ref26] An S.; Suh Y.; Kelich P.; Lee D.; Vukovic L.; Jeong S. Directed Evolution of Near-Infrared Serotonin Nanosensors with Machine Learning-Based Screening. Nanomaterials 2024, 14 (3), 247 10.3390/nano14030247. 38334518 PMC10856788

[ref27] Beyene A. G.; Delevich K.; Del Bonis-O’Donnell J. T.; Piekarski D. J.; Lin W. C.; Thomas A. W.; Yang S. J.; Kosillo P.; Yang D.; Prounis G. S.; Wilbrecht L.; Landry M. P. Imaging striatal dopamine release using a nongenetically encoded near infrared fluorescent catecholamine nanosensor. Sci. Adv. 2019, 5 (7), eaaw3108 10.1126/sciadv.aaw3108. 31309147 PMC6620097

[ref28] Bulumulla C.; Krasley A. T.; Cristofori-Armstrong B.; Valinsky W. C.; Walpita D.; Ackerman D.; Clapham D. E.; Beyene A. G. Visualizing Synaptic Dopamine Efflux with a 2D Composite Nanofilm. eLife 2022, 11, e78773 10.7554/eLife.78773. 35786443 PMC9363124

[ref29] Kruss S.; Salem D. P.; Vuković L.; Lima B.; Vander Ende E.; Boyden E. S.; Strano M. S. High-resolution imaging of cellular dopamine efflux using a fluorescent nanosensor array. Proc. Natl. Acad. Sci. U.S.A. 2017, 114 (8), 1789–1794. 10.1073/pnas.1613541114. 28179565 PMC5338365

[ref30] Elizarova S.; Chouaib A. A.; Shaib A.; Hill B.; Mann F.; Brose N.; Kruss S.; Daniel J. A. A fluorescent nanosensor paint detects dopamine release at axonal varicosities with high spatiotemporal resolution. Proc. Natl. Acad. Sci. U.S.A. 2022, 119 (22), e2202842119 10.1073/pnas.2202842119. 35613050 PMC9295782

[ref31] Kilian P.; Knight F. R.; Woollins J. D. Synthesis of ligands based on naphthalene peri-substituted by Group 15 and 16 elements and their coordination chemistry. Coord. Chem. Rev. 2011, 255 (11–12), 1387–1413. 10.1016/j.ccr.2011.01.015.

[ref32] Pelizzetti E.; Mentasti E. Kinetics and Mechanism of Oxidation of Catechols by Tris(1,10-Phenanthroline) Iron (III) and Its Derivatives in Aqueous Acidic Perchlorate Media. Zeitschrift für Physikalische Chemie 1977, 105 (1–2), 21–34. 10.1524/zpch.1977.105.1_2.021.

[ref33] Bergquist J.; Ściubisz A.; Kaczor A.; Silberring J. Catecholamines and methods for their identification and quantitation in biological tissues and fluids. Journal of Neuroscience Methods 2002, 113 (1), 1–13. 10.1016/S0165-0270(01)00502-7. 11741716

[ref34] Tsunoda M. Recent advances in methods for the analysis of catecholamines and their metabolites. Anal. Bioanal. Chem. 2006, 386 (3), 506–514. 10.1007/s00216-006-0675-z. 16924378

[ref35] Ribeiro J. A.; Fernandes P. M. V.; Pereira C. M.; Silva F. Electrochemical sensors and biosensors for determination of catecholamine neurotransmitters: A review. Talanta 2016, 160, 653–679. 10.1016/j.talanta.2016.06.066. 27591662

[ref36] Barone P. W.; Baik S.; Heller D. A.; Strano M. S. Near-infrared optical sensors based on single-walled carbon nanotubes. Nat. Mater. 2005, 4 (1), 86–92. 10.1038/nmat1276. 15592477

[ref37] Satishkumar B. C.; Brown L. O.; Gao Y.; Wang C. C.; Wang H. L.; Doorn S. K. Reversible fluorescence quenching in carbon nanotubes for biomolecular sensing. Nat. Nanotechnol. 2007, 2 (9), 560–564. 10.1038/nnano.2007.261. 18654368

[ref38] Yamabe S.; Minato T.; Kimura M. Theoretical interpretation of the standard redox potential of benzene-1,2-diol and its derivatives. J. Phys. Chem. 1981, 85 (23), 3510–3513. 10.1021/j150623a029.

[ref39] Jaramillo A. M.; Barrera-Gutiérrez R.; Cortés M. T. Synthesis, Follow-Up, and Characterization of Polydopamine-like Coatings Departing from Micromolar Dopamine-<i > o</i>-Quinone Precursor Concentrations. ACS Omega 2020, 5 (25), 15016–15027. 10.1021/acsomega.0c00676. 32637775 PMC7330902

[ref40] Zhang Z.; He X.; Zhou C.; Reaume M.; Wu M.; Liu B.; Lee B. P. Iron Magnetic Nanoparticle-Induced ROS Generation from Catechol-Containing Microgel for Environmental and Biomedical Applications. ACS Applied Materials &amp; Interfaces 2020, 12 (19), 21210–21220. 10.1021/acsami.9b19726. 32069006 PMC7228842

[ref41] O’Connell M. J.; Bachilo S. M.; Huffman C. B.; Moore V. C.; Strano M. S.; Haroz E. H.; Rialon K. L.; Boul P. J.; Noon W. H.; Kittrell C.; Ma J.; Hauge R. H.; Weisman R. B.; Smalley R. E. Band Gap Fluorescence from Individual Single-Walled Carbon Nanotubes. Science 2002, 297 (5581), 593–596. 10.1126/science.1072631. 12142535

[ref42] Duque J. G.; Cognet L.; Parra-Vasquez A. N. G.; Nicholas N.; Schmidt H. K.; Pasquali M. Stable Luminescence from Individual Carbon Nanotubes in Acidic, Basic, and Biological Environments. J. Am. Chem. Soc. 2008, 130 (8), 2626–2633. 10.1021/ja0777234. 18237169

[ref43] Chemicalize, https://chemicalize.com/ (accessed April 26, 2024).

[ref44] Silvera-Batista C. A.; Wang R. K.; Weinberg P.; Ziegler K. J. Solvatochromic shifts of single-walled carbon nanotubes in nonpolar microenvironments. Phys. Chem. Chem. Phys. 2010, 12 (26), 6990 10.1039/b927053a. 20463994

[ref45] Song C.; Pehrsson P. E.; Zhao W. Recoverable Solution Reaction of HiPco Carbon Nanotubes with Hydrogen Peroxide. J. Phys. Chem. B 2005, 109 (46), 21634–21639. 10.1021/jp053077o. 16853809

[ref46] Jena P. V.; Roxbury D.; Galassi T. V.; Akkari L.; Horoszko C. P.; Iaea D. B.; Budhathoki-Uprety J.; Pipalia N.; Haka A. S.; Harvey J. D.; Mittal J.; Maxfield F. R.; Joyce J. A.; Heller D. A. A Carbon Nanotube Optical Reporter Maps Endolysosomal Lipid Flux. ACS Nano 2017, 11 (11), 10689–10703. 10.1021/acsnano.7b04743. 28898055 PMC5707631

[ref47] Budhathoki-Uprety J.; Shah J.; Korsen J. A.; Wayne A. E.; Galassi T. V.; Cohen J. R.; Harvey J. D.; Jena P. V.; Ramanathan L. V.; Jaimes E. A.; Heller D. A. Synthetic molecular recognition nanosensor paint for microalbuminuria. Nature Communications 2019 10:1 2019, 10 (1), 1–9. 10.1038/s41467-019-11583-1. PMC6689023 31399600

[ref48] Bisker G.; Dong J.; Park H. D.; Iverson N. M.; Ahn J.; Nelson J. T.; Landry M. P.; Kruss S.; Strano M. S. Protein-targeted corona phase molecular recognition. Nat. Commun. 2016, 7 (1), 1–14. 10.1038/ncomms10241. PMC4729864 26742890

[ref49] Gerstman E.; Hendler-Neumark A.; Wulf V.; Bisker G. Monitoring the Formation of Fibrin Clots as Part of the Coagulation Cascade Using Fluorescent Single-Walled Carbon Nanotubes. ACS Appl. Mater. Interfaces 2023, 15, 21866–21876. 10.1021/acsami.3c00828. 37128896 PMC10176323

[ref50] Williams R. M.; Lee C.; Galassi T. V.; Harvey J. D.; Leicher R.; Sirenko M.; Dorso M. A.; Shah J.; Olvera N.; Dao F.; Levine D. A.; Heller D. A. Noninvasive ovarian cancer biomarker detection via an optical nanosensor implant. Sci. Adv. 2018, 4 (4), eaaq1090 10.1126/sciadv.aaq1090. 29675469 PMC5906074

[ref51] Antman-Passig M.; Wong E.; Frost G. R.; Cupo C.; Shah J.; Agustinus A.; Chen Z.; Mancinelli C.; Kamel M.; Li T.; Jonas L. A.; Li Y.-M.; Heller D. A. Optical Nanosensor for Intracellular and Intracranial Detection of Amyloid-Beta. ACS Nano 2022, 16 (5), 7269–7283. 10.1021/acsnano.2c00054. 35420796 PMC9710299

[ref52] Pinals R. L.; Ledesma F.; Yang D.; Navarro N.; Jeong S.; Pak J. E.; Kuo L.; Chuang Y. C.; Cheng Y. W.; Sun H. Y.; Landry M. P. Rapid SARS-CoV-2 Spike Protein Detection by Carbon Nanotube-Based Near-Infrared Nanosensors. Nano Lett. 2021, 21 (5), 2272–2280. 10.1021/acs.nanolett.1c00118. 33635655 PMC10493163

[ref53] Nißler R.; Bader O.; Dohmen M.; Walter S. G.; Noll C.; Selvaggio G.; Groß U.; Kruss S. Remote near infrared identification of pathogens with multiplexed nanosensors. Nat. Commun. 2020, 11 (1), 5995 10.1038/s41467-020-19718-5. 33239609 PMC7689463

[ref54] Polo E.; Kruss S. Impact of Redox-Active Molecules on the Fluorescence of Polymer-Wrapped Carbon Nanotubes. J. Phys. Chem. C 2016, 120 (5), 3061–3070. 10.1021/acs.jpcc.5b12183.

[ref55] Kurnosov N. V.; Leontiev V. S.; Linnik A. S.; Lytvyn O. S.; Karachevtsev V. A. Photoluminescence intensity enhancement in SWNT aqueous suspensions due to reducing agent doping: Influence of adsorbed biopolymer. Chem. Phys. 2014, 438, 23–30. 10.1016/j.chemphys.2014.04.006.

[ref56] Chang C. J.; James T. D.; New E. J.; Tang B. Z. Activity-Based Sensing: Achieving Chemical Selectivity through Chemical Reactivity. Acc. Chem. Res. 2020, 53 (1), 1–1. 10.1021/acs.accounts.9b00542. 31958959

[ref57] Messina M. S.; Quargnali G.; Chang C. J. Activity-Based Sensing for Chemistry-Enabled Biology: Illuminating Principles, Probes, and Prospects for Boronate Reagents for Studying Hydrogen Peroxide. ACS Bio &amp; Med. Chem. Au 2022, 2 (6), 548–564. 10.1021/acsbiomedchemau.2c00052. PMC9782337 36573097

[ref58] Wheeler S. E. Understanding Substituent Effects in Noncovalent Interactions Involving Aromatic Rings. Acc. Chem. Res. 2013, 46 (4), 1029–1038. 10.1021/ar300109n. 22725832

[ref59] Dennington R.; Keith T.; Millam J. GaussView, 6.1.1; Semichem Inc.: Shawnee Mission, KS, 2019.

[ref60] Hart K.; Foloppe N.; Baker C. M.; Denning E. J.; Nilsson L.; Mackerell A. D. Optimization of the CHARMM Additive Force Field for DNA: Improved Treatment of the BI/BII Conformational Equilibrium. J. Chem. Theory Comput. 2012, 8 (1), 348–362. 10.1021/ct200723y. 22368531 PMC3285246

[ref61] Huang J.; Mackerell A. D. CHARMM36 all-atom additive protein force field: Validation based on comparison to NMR data. J. Comput. Chem. 2013, 34 (25), 2135–2145. 10.1002/jcc.23354. 23832629 PMC3800559

[ref62] Zheng Y.; Alizadehmojarad A. A.; Bachilo S. M.; Kolomeisky A. B.; Weisman R. B. Dye Quenching of Carbon Nanotube Fluorescence Reveals Structure-Selective Coating Coverage. ACS Nano 2020, 14 (9), 12148–12158. 10.1021/acsnano.0c05720. 32845604

[ref63] Alizadehmojarad A. A.; Bachilo S. M.; Weisman R. B. Compositional Analysis of ssDNA-Coated Single-Wall Carbon Nanotubes through UV Absorption Spectroscopy. Nano Lett. 2022, 22 (20), 8203–8209. 10.1021/acs.nanolett.2c02850. 36201880

[ref64] Alizadehmojarad A. A.; Zhou X.; Beyene A. G.; Chacon K. E.; Sung Y.; Pinals R. L.; Landry M. P.; Vuković L. Binding Affinity and Conformational Preferences Influence Kinetic Stability of Short Oligonucleotides on Carbon Nanotubes. Advanced Materials Interfaces 2020, 7 (15), 2000353 10.1002/admi.202000353.

[ref65] Nißler R.; Mann F. A.; Chaturvedi P.; Horlebein J.; Meyer D.; Vuković L.; Kruss S. Quantification of the Number of Adsorbed DNA Molecules on Single-Walled Carbon Nanotubes. J. Phys. Chem. C 2019, 123 (8), 4837–4847. 10.1021/acs.jpcc.8b11058.

[ref66] Vanommeslaeghe K.; Hatcher E.; Acharya C.; Kundu S.; Zhong S.; Shim J.; Darian E.; Guvench O.; Lopes P.; Vorobyov I.; Mackerell A. D. CHARMM general force field: A force field for drug-like molecules compatible with the CHARMM all-atom additive biological force fields. J. Comput. Chem. 2010, 31 (4), 671–690. 10.1002/jcc.21367. 19575467 PMC2888302

[ref67] Phillips J. C.; Hardy D. J.; Maia J. D. C.; Stone J. E.; Ribeiro J. V.; Bernardi R. C.; Buch R.; Fiorin G.; Hénin J.; Jiang W.; McGreevy R.; Melo M. C. R.; Radak B. K.; Skeel R. D.; Singharoy A.; Wang Y.; Roux B.; Aksimentiev A.; Luthey-Schulten Z.; Kalé L. V.; Schulten K.; Chipot C.; Tajkhorshid E. Scalable molecular dynamics on CPU and GPU architectures with NAMD. J. Chem. Phys. 2020, 153 (4), 044130 10.1063/5.0014475. 32752662 PMC7395834

[ref68] Darden T.; York D.; Pedersen L. Particle mesh Ewald: An < i > N</i>·log(<i > N</i>) method for Ewald sums in large systems. J. Chem. Phys. 1993, 98 (12), 10089–10092. 10.1063/1.464397.

[ref69] Sánchez H. R. Residence Times from Molecular Dynamics Simulations. J. Phys. Chem. B 2022, 126 (43), 8804–8812. 10.1021/acs.jpcb.2c03756. 36269165

[ref70] Bernetti M.; Masetti M.; Rocchia W.; Cavalli A. Kinetics of Drug Binding and Residence Time. Annu. Rev. Phys. Chem. 2019, 70 (1), 143–171. 10.1146/annurev-physchem-042018-052340. 30786217

[ref71] Pan A. C.; Xu H.; Palpant T.; Shaw D. E. Quantitative Characterization of the Binding and Unbinding of Millimolar Drug Fragments with Molecular Dynamics Simulations. J. Chem. Theory Comput. 2017, 13 (7), 3372–3377. 10.1021/acs.jctc.7b00172. 28582625

[ref72] Krasley A.; Chakraborty S.; Vukovic L.; Beyene A. Molecular Determinants of Optical Modulation in ssDNA-Carbon Nanotube Biosensors: Insights from Experimental and Computational Approaches. 2024, *ChemRxiv*, https://chemrxiv.org/engage/chemrxiv/article-details/66e9f8e2cec5d6c1425f7a78 (accessed September 18, 2024).

