# Supplementary material for: Molecular Determinants of Optical Modulation in ssDNA–Carbon Nanotube Biosensors
Source: ACS Nano. 2025 Jan 16;19(8):7804–20. doi: 10.1021/acsnano.4c13814 (PMC11887485; doi:10.1021/acsnano.4c13814)
Supplement: Supplementary file 1 — nn4c13814_si_001.pdf [file nn4c13814_si_001.pdf]

Supplementary Information for

Molecular Determinants of Optical Modulation in ssDNA-Carbon Nanotube Biosensors

Andrew T. Krasley[1,†], Sayantani Chakraborty[2,†], Lela Vuković[2,3,*], Abraham G. Beyene[1,*]

[1] Janelia Research Campus, Howard Hughes Medical Institute, Ashburn, VA 20147

[2] Department of Chemistry and Biochemistry, University of Texas at El Paso, El Paso, TX 79968

[3] Computational Science Program and Bioinformatics Program, University of Texas at El Paso, El Paso, TX 79968

* Corresponding authors. Email: lvukovic@utep.edu; beyenea@janelia.hhmi.org

† These authors contributed equally to this work.

Supplementary Figures

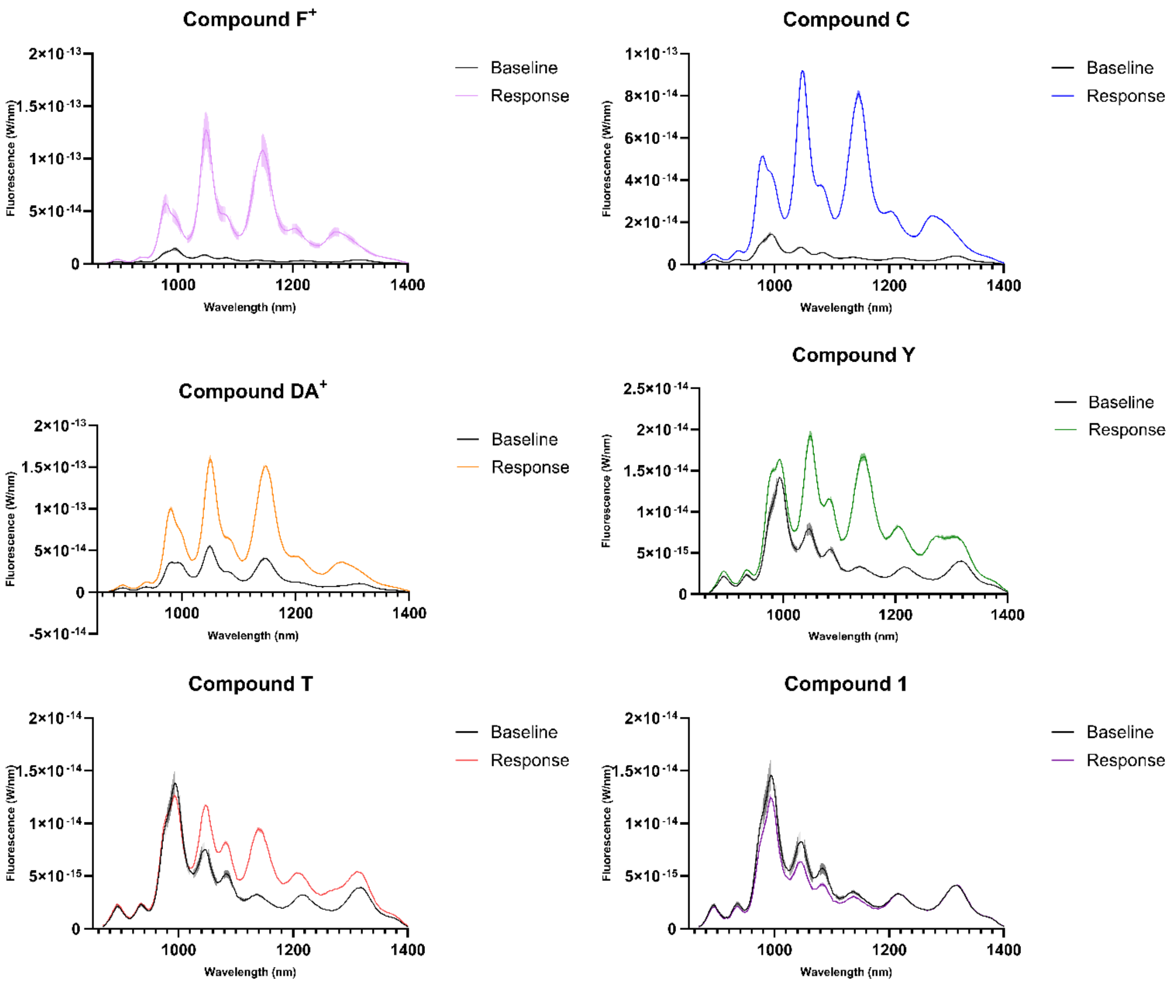

Figure S1. Example of baseline and response fluorescence spectra for six compounds. Experimental fluorescence measurements of the (GT)$_6$-SWCNT suspension were taken 30 min after the addition of compounds (10 μM final concentration). All readings (n = 3) were taken with 10 ppm (GT)$_6$-SWCNT in 0.1M NaCl with a 658 nm excitation laser, at 52.4 mW power. Ligands were added from DMSO stock solutions (Methods). Charts display solid lines as mean with one standard deviation band. Note that **DA**$^+$ and **F**$^+$ exist in their protonated state under these experimental conditions.

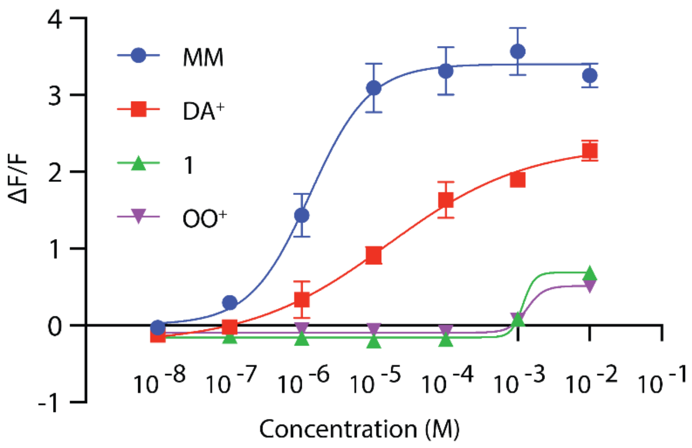

Figure S2. **Dose Response Curves.** Experimental fluorescence measurements of the $(GT)_6$-SWCNT suspension were taken after the addition of compounds at varying concentations (10 nM to 10 mM final concentrations). Responses are consistent from 10 nM to 10 mM (**MM** > **DA**$^+$ > **1** > **OO**). The Hill equation was used to fit the experimental data..

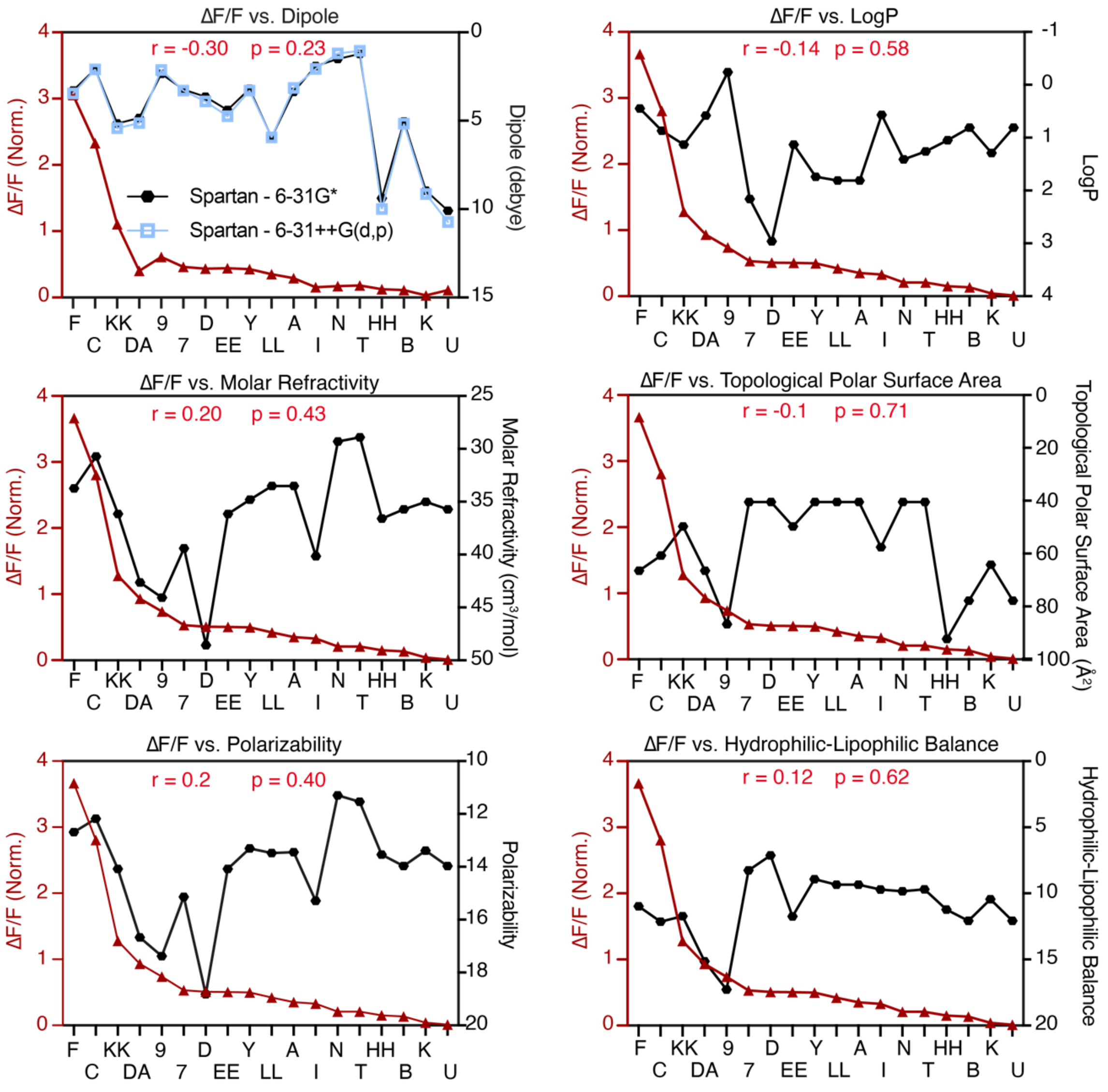

Figure S3. Plots of ΔF/F for a subset of 18 compounds vs. various electronic, physical, and calculated properties. See Figure 2a for compound structures. No correlation was observed in these six charts. Dipole values were computed using Spartan'20 V1.1.14 on minimized structures using equilibrium geometry at ground state in water with density functional B3LYP and 6-31G* (black filled circles) and 6-31++G(d,p) (blue open squares) basis sets. LogP, molar refractivity, and topological polar surface area were calculated using Chemdraw 22.0.0. Polarizability and hydrophilic-lipophilic balance were calculated using Chemaxon Chemicalize.

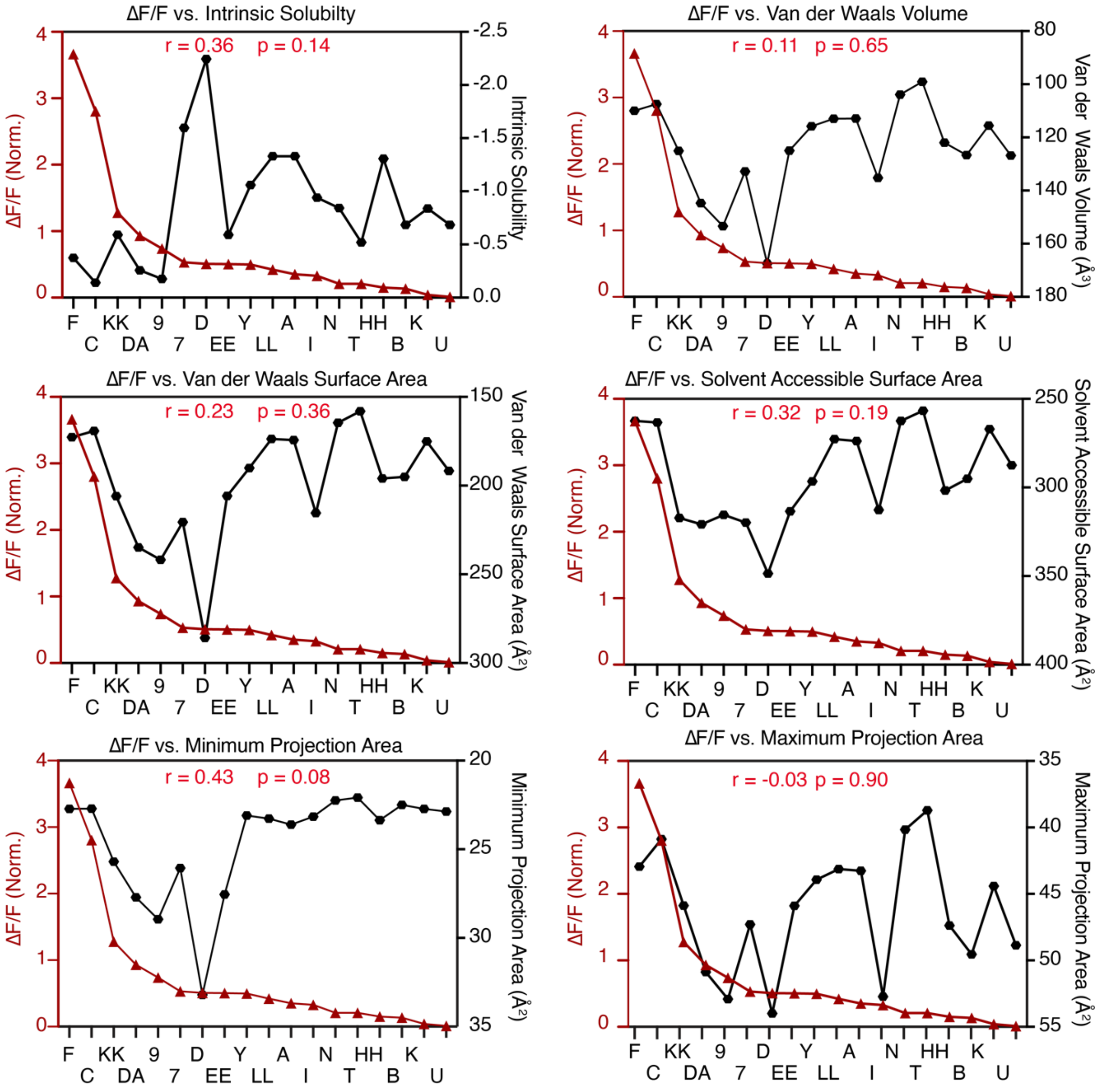

Figure S4. Plots of ΔF/F for a subset of 18 compounds vs. various electronic, physical, and calculated properties. See Figure 2a for compound structures. No correlation was observed in these six charts. Intrinsic solubility, van der Waals volume and surface area, solvent accessible surface area, and minimum and maximum projection areas were all calculated using Chemaxon Chemicalize.

a

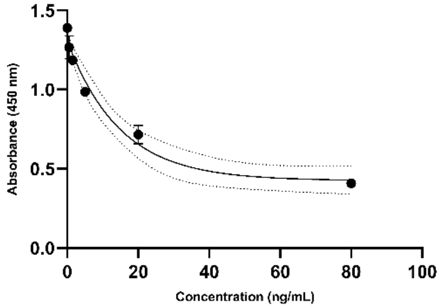

	Acceptance Range (ng/mL)	Sample Value (ng/mL)
QC1	1.8-4.2	4.1
QC2	6.0-14	13

b

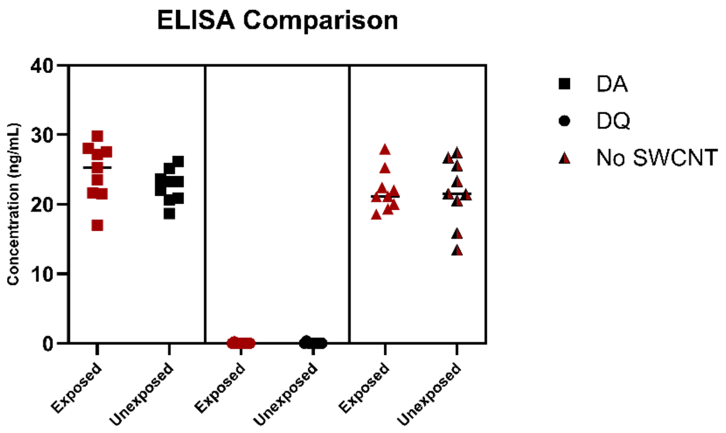

c

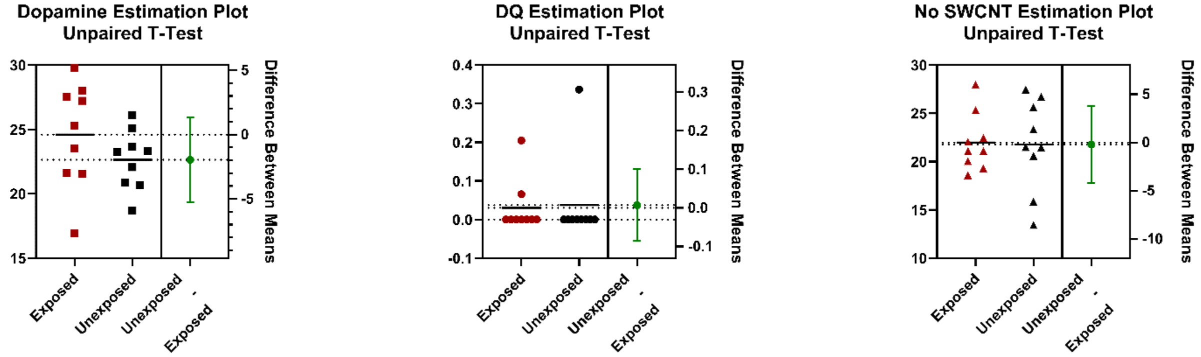

Figure S5. Dopamine ELISA results showing no difference between samples exposed and unexposed to 658 nm light. This lack of difference between exposed and unexposed samples indicates that the same level of dopamine is present in both. The ELISA kit (ImmuSmol SAS, Bordeaux, France) was run experimentally and processed in accordance with the manufacture's guidelines and standard operating procedure. It has a functional sensitivity of 5 pg/mL and a LOD of 3.3 pg/mL. **a)** Standard curve used for interpolation and QC results. **b)** Comparison of interpolated results. DA = dopamine, DQ = dopaquinone, No SWCNT = samples run without SWCNT present in solution. **c)** Unpaired t-tests showing no significant difference between exposed and unexposed populations of each.

Figure S6. HPLC conditions and spectra.

Solvent A: 95:5 H_2O:ACN + 30 mM Ammonium Formate
Solvent B: 15:85 H_2O:ACN + 30 mM Ammonium Formate

Time (min)	Flow (mL/min)	%A	%B
0.00	2.00	0	100
0.80	2.00	0	100
3.00	2.00	10	90
6.00	2.00	10	90
10.00	2.00	0	100

Sample Temp.: 8°C
Column Temp.: 40°C
Injection Volume: 1.0 µL
Sample Matrix: H_2O
PDA wavelength: 280 nm
HPLC: Waters Arc Premier
Column: 4.6 x 50 mm x 2.5 µm XBridge Premier BEH Amide with VanGuard

S A M P L E   I N F O R M A T I O N

Sample Name:	DA	Acquired By:	System
Sample Type:	Unknown	Processing Method	10 min processing
Vial:	1:A,2	Channel Name:	2998 Ch6 280nm@4.8nm
Injection #:	1	Proc. Chnl. Descr.:	2998 Ch6 280nm@4.8nm
Injection Volume:	1.00 ul		
Run Time:	10.0 Minutes		
Date Acquired:	5/25/2023 12:59:28 PM EDT		

Auto-Scaled Chromatogram

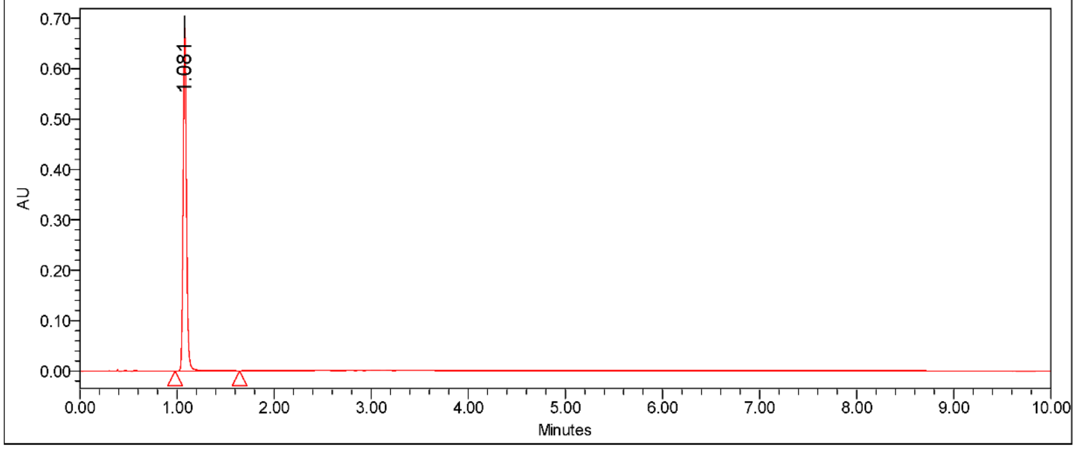

Peak Results

	RT	Area	Height	% Area
1	1.081	1637723	685415	100.00

SAMPLE INFORMATION

Sample Name:	DQ	Acquired By:	System
Sample Type:	Unknown	Processing Method	10 min processing
Vial:	1:A,6	Channel Name:	2998 Ch6 280nm@4.8nm
Injection #:	1	Proc. Chnl. Descr.:	2998 Ch6 280nm@4.8nm
Injection Volume:	1.00 ul		
Run Time:	10.0 Minutes		
Date Acquired:	5/25/2023 2:33:53 PM EDT		

Auto-Scaled Chromatogram

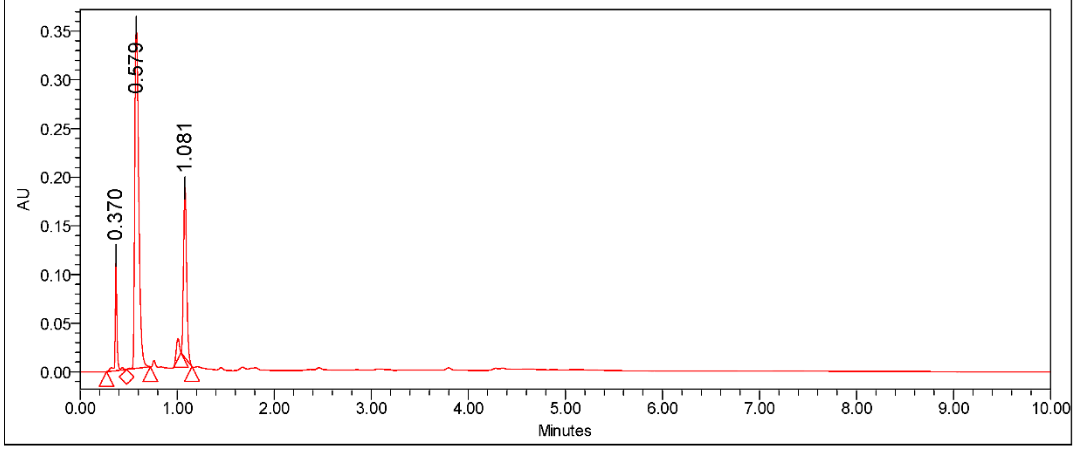

Peak Results

	RT	Area	Height	% Area
1	0.370	143723	118945	9.35
2	0.579	1001073	351106	65.15
3	1.081	391761	175183	25.50

SAMPLE INFORMATION

Sample Name:	Exposed	Acquired By:	System
Sample Type:	Unknown	Processing Method	10 min processing
Vial:	1:A,7	Channel Name:	2998 Ch6 280nm@4.8nm
Injection #:	1	Proc. Chnl. Descr.:	2998 Ch6 280nm@4.8nm
Injection Volume:	1.00 ul		
Run Time:	10.0 Minutes		
Date Acquired:	5/25/2023 1:23:16 PM EDT		

Auto-Scaled Chromatogram

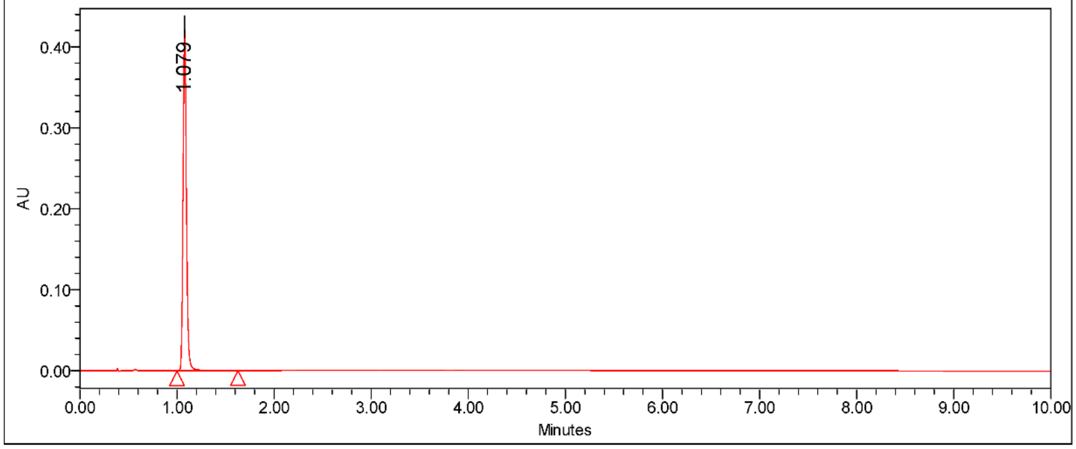

Peak Results

	RT	Area	Height	% Area
1	1.079	1035589	426262	100.00

SAMPLE INFORMATION

Sample Name:	H2O Blank	Acquired By:	System
Sample Type:	Unknown	Processing Method	10 min processing
Vial:	1:A,1	Channel Name:	2998 Ch6 280nm@4.8nm
Injection #:	1	Proc. Chnl. Descr.:	2998 Ch6 280nm@4.8nm
Injection Volume:	1.00 ul		
Run Time:	10.0 Minutes		
Date Acquired:	5/25/2023 12:47:35 PM EDT		

Auto-Scaled Chromatogram

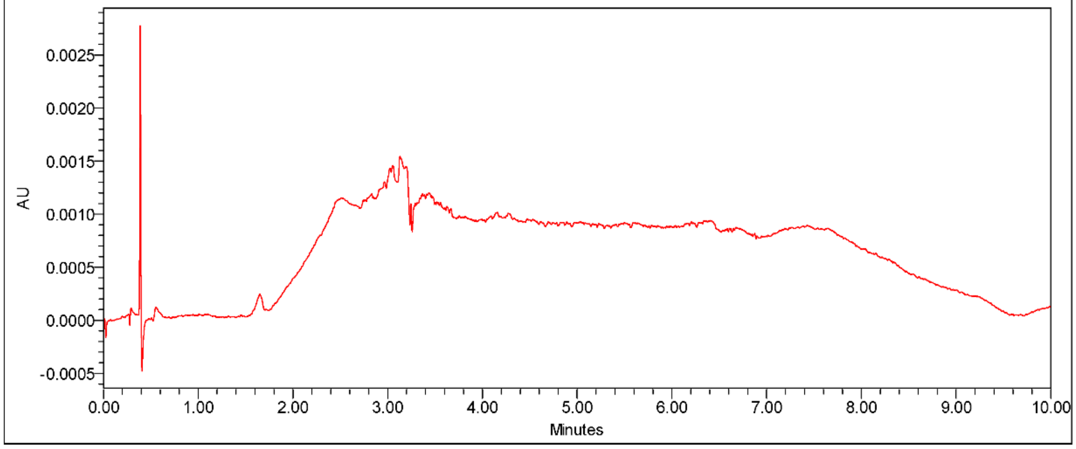

Peak Results

	RT	Area	Height	% Area
1				

Reported by User: System
Report Method: Default
Report Method ID: 23653
Page: 1 of 1

Date Printed:
3/21/2024
10:45:29 AM US/Eastern

SAMPLE INFORMATION

Sample Name:	NaIO4	Acquired By:	System
Sample Type:	Unknown	Processing Method	10 min processing
Vial:	1:A,3	Channel Name:	2998 Ch6 280nm@4.8nm
Injection #:	1	Proc. Chnl. Descr.:	2998 Ch6 280nm@4.8nm
Injection Volume:	1.00 ul		
Run Time:	10.0 Minutes		
Date Acquired:	5/25/2023 1:47:03 PM EDT		

Auto-Scaled Chromatogram

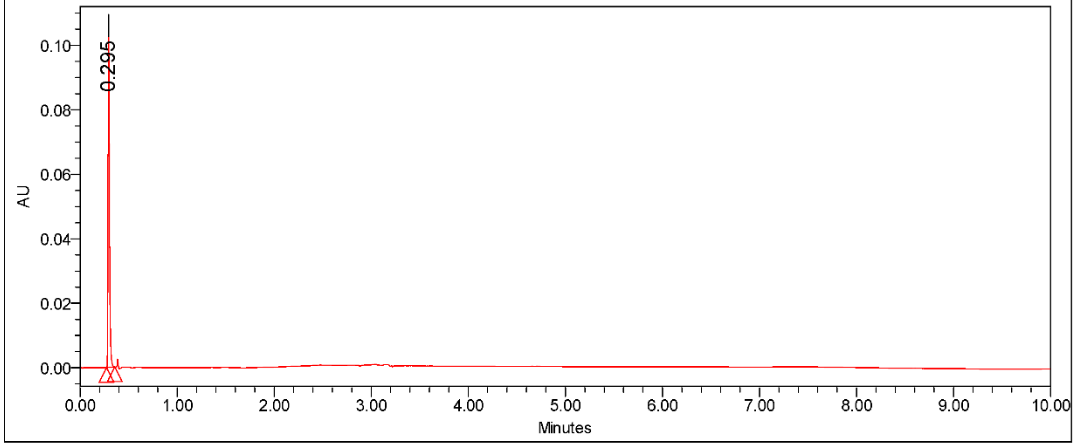

Peak Results

	RT	Area	Height	% Area
1	0.295	104084	106431	100.00

SAMPLE INFORMATION

Sample Name:	Unexposed	Acquired By:	System
Sample Type:	Unknown	Processing Method	10 min processing
Vial:	1:A,8	Channel Name:	2998 Ch6 280nm@4.8nm
Injection #:	1	Proc. Chnl. Descr.:	2998 Ch6 280nm@4.8nm
Injection Volume:	1.00 ul		
Run Time:	10.0 Minutes		
Date Acquired:	5/25/2023 1:35:09 PM EDT		

Auto-Scaled Chromatogram

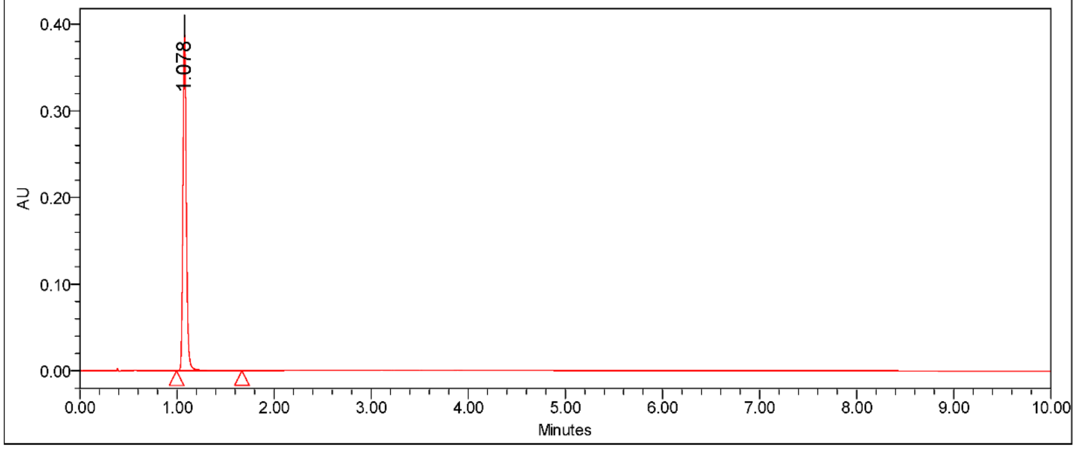

Peak Results

	RT	Area	Height	% Area
1	1.078	965002	397922	100.00

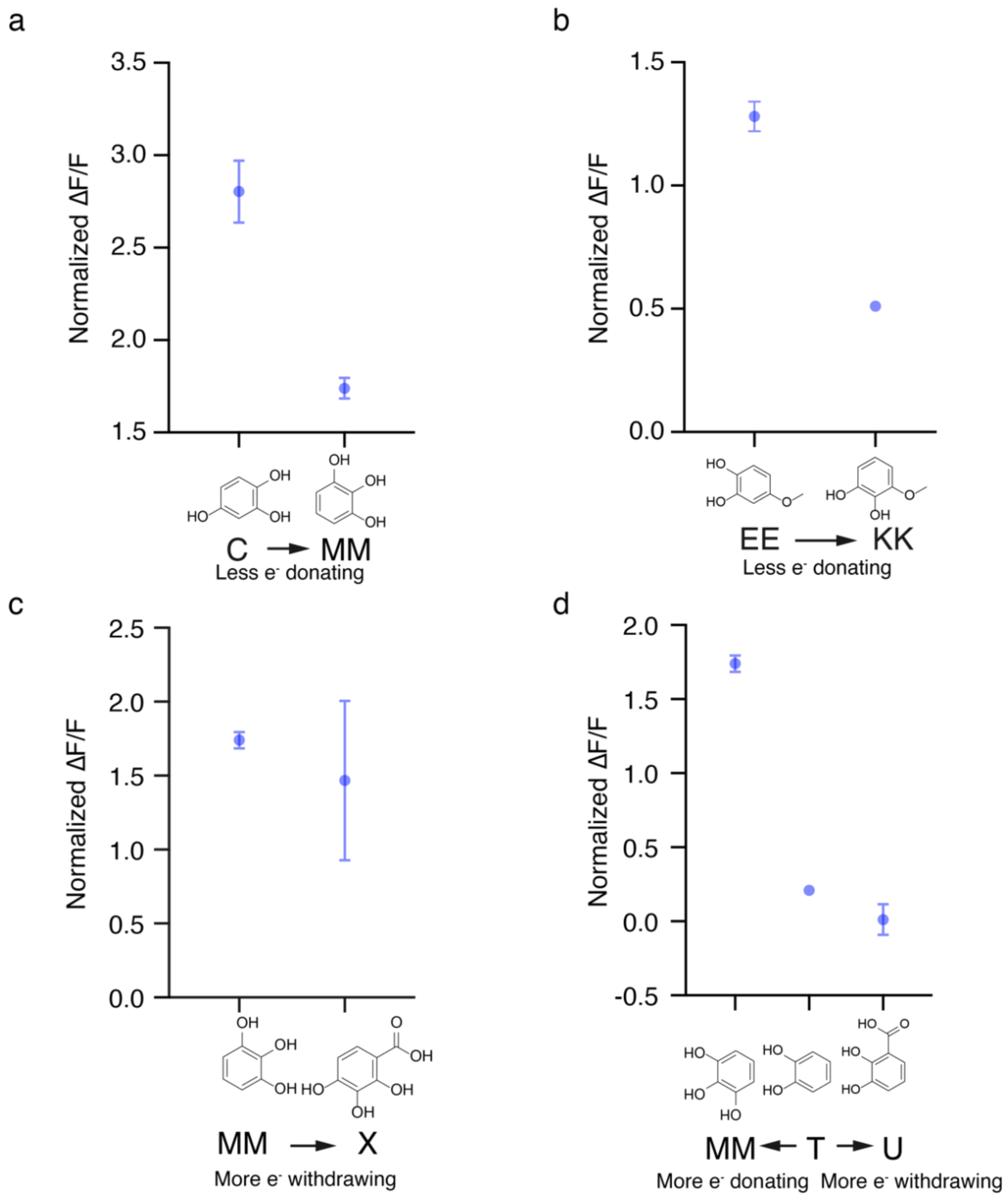

Figure S7. Comparative analysis of normalized change in fluorescence to electron donating and withdrawing substituents on benzene-1,2-diols. Trends demonstrate that more electron donating substitutes or less electron withdrawing substitutes produce a larger change in fluorescence than their respective less donating or more withdrawing counterparts. Normalization was relative to dopamine = 1.00. **a)** When a hydroxy is in the 4-position (**C**), it is more electron donating than when a hydroxy is in the 3-position (**MM**), and this is reflected in larger change in fluorescence. **b)** In the same manner as **(a)**, the methoxy in the 4-position (**KK**) is more electron donating than when in the 3-position (**EE**), leading to a higher change in fluorescence. **c)** When looking at isomers where electron withdrawing groups are added, the compound containing the electron withdrawing group (carboxyl in **X**) produces a lower change in fluorescence than its isomer without the withdrawing group (**MM**). **d)** When applying this concept to benzene-1,2-diols that have a donating group (**MM**) or withdrawing group (**U**) relative to benzene-1,2-diol (**T**) the same trend can be observed, with more donating producing a larger change in fluorescence than the withdrawing substituents. Mean and standard deviation values are shown in all charts.

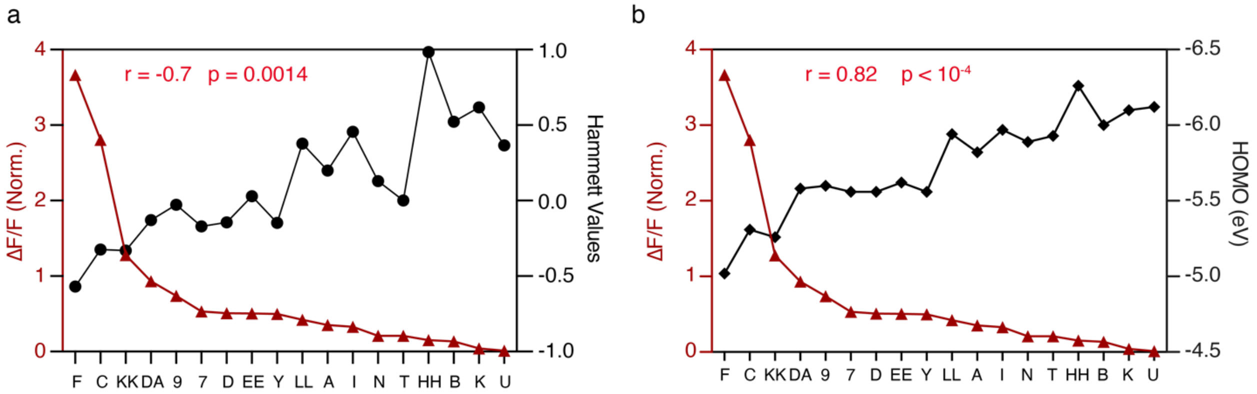

Figure S8. Correlations between optical modulations and Hammett values or computationally determined eHOMO levels. a) Correlational analysis of change in fluorescence for each compound (mean, normalized relative to dopamine = 1.00) vs. their calculated Hammett values showing a significant correlation (Pearson statistic and p-value are shown). This is a complementary presentation of the data presented in Figure 2C. **b)** Correlational analysis of change in fluorescence for each compound (normalized relative to dopamine = 1.00) vs. their calculated HOMO level showing a significant correlation (Pearson statistic and p-value are shown). All HOMO values were calculated using Spartan'20 V1.1.14 on minimized structures using equilibrium geometry at ground state in water with density functional B3LYP 6-31G*.

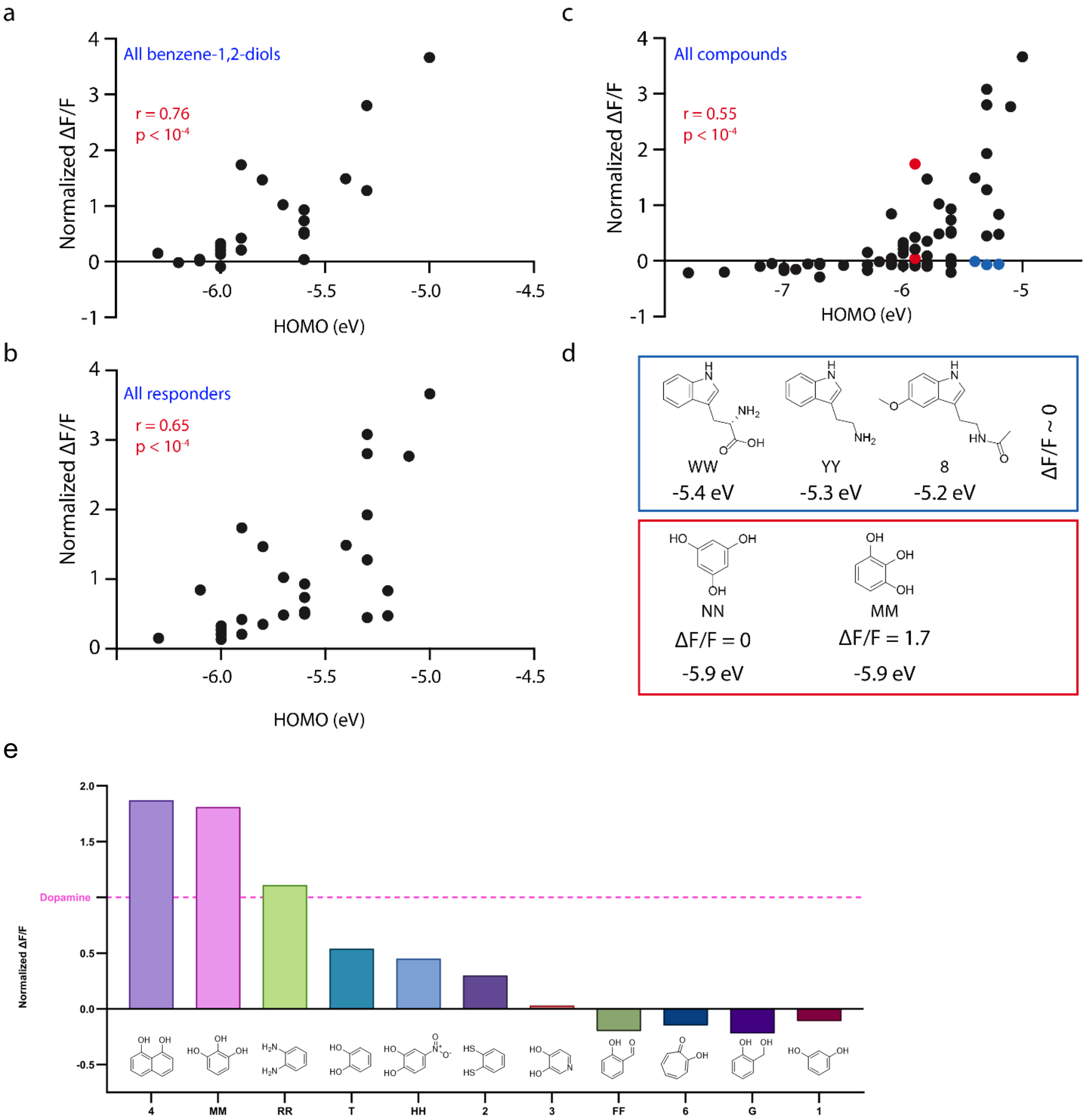

Figure S9. Correlational analysis of change in fluorescence for each compound vs. their calculated HOMO level. All compounds mean values normalized to dopamine = 1.00. **a)** Analysis of all benzene-1,2-diols showing a positive correlation. **b)** Analysis of all responsive compounds (groups I, II and III in Figure 1c) showing a positive correlation. **c)** Analysis of all compounds showing a positive correlation. **d)** HOMO level alone is not enough to predict responsiveness. Blue box shows three compounds with favorable HOMO levels but elicit no optical response. Red box shows two compounds with the same HOMO levels but different optical responses. The colored boxes represent the respective compounds depicted in the same color in (**c**). **e)** Trend showing that vicinal hydrogen bond donors are needed for a response and that more electron donating rich produce a higher response. All HOMO values were calculated using Spartan'20 V1.1.14 on minimized structures using equilibrium geometry at ground state in water with density functional B3LYP 6-31G*.

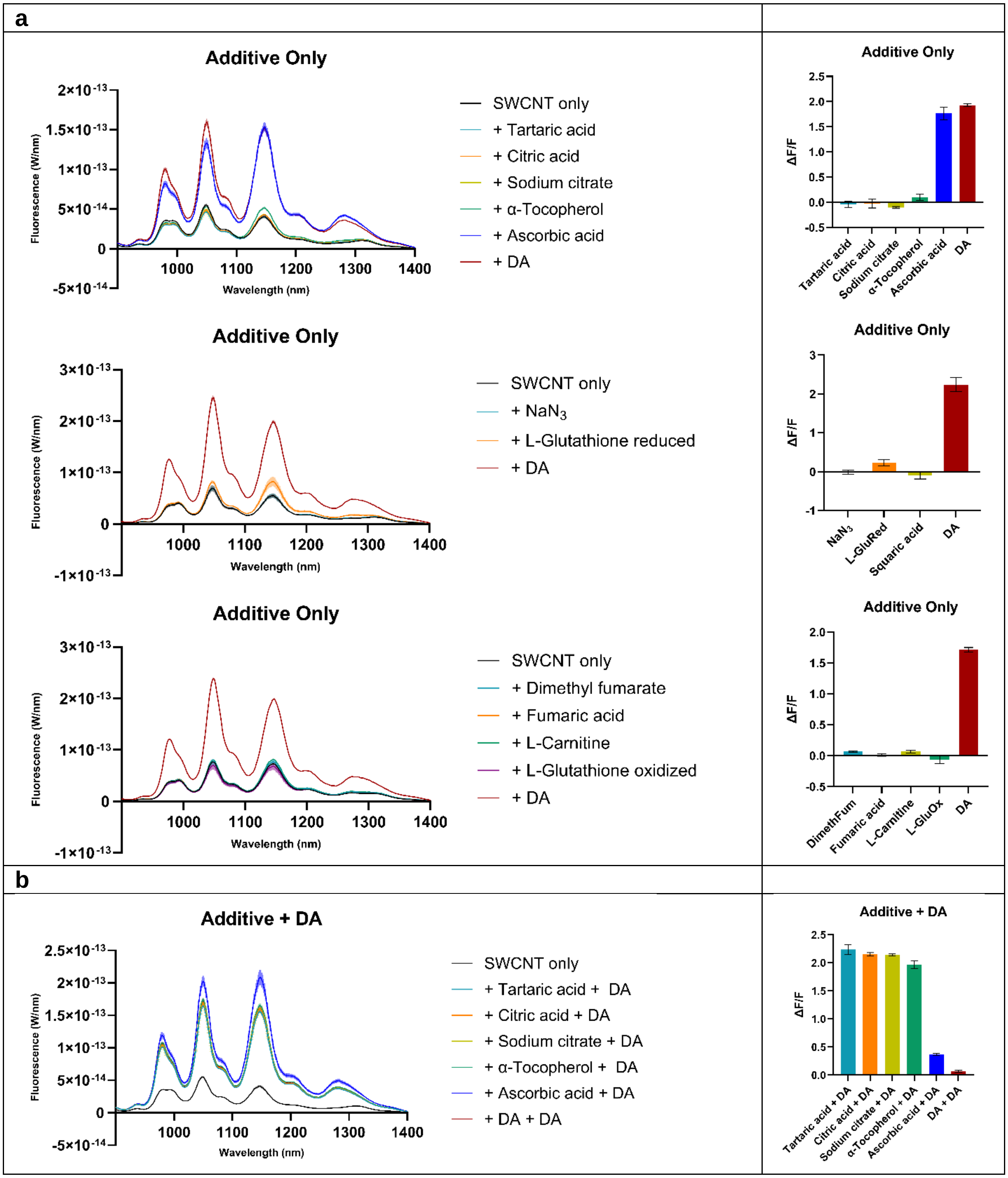
a
Additive Only
Fluorescence (W/nm)
Wavelength (nm)
SWCNT only
+ Tartaric acid
+ Citric acid
+ Sodium citrate
+ α-Tocopherol
+ Ascorbic acid
+ DA
ΔF/F
Tartaric acid
Citric acid
Sodium citrate
α-Tocopherol
Ascorbic acid
DA
Additive Only
SWCNT only
+ NaN3
+ L-Glutathione reduced
+ DA
NaN3
L-GluRed
Squaric acid
DA
Additive Only
SWCNT only
+ Dimethyl fumarate
+ Fumaric acid
+ L-Carnitine
+ L-Glutathione oxidized
+ DA
DimethFum
Fumaric acid
L-Carnitine
L-GluOx
DA
b
Additive + DA
SWCNT only
+ Tartaric acid + DA
+ Citric acid + DA
+ Sodium citrate + DA
+ α-Tocopherol + DA
+ Ascorbic acid + DA
+ DA + DA
Tartaric acid + DA
Citric acid + DA
Sodium citrate + DA
α-Tocopherol + DA
Ascorbic acid + DA
DA + DA

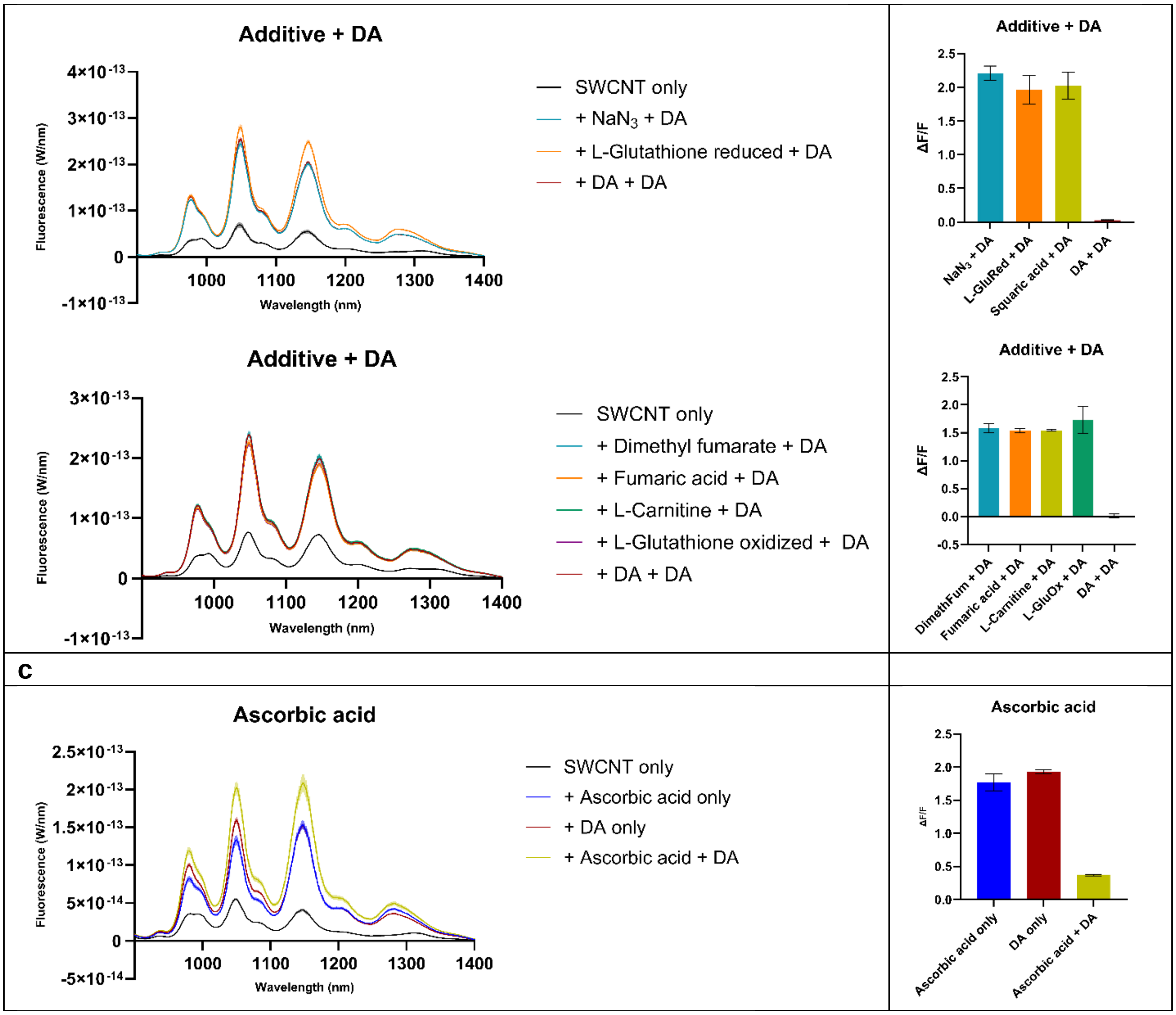

Figure S10. The effect of reducing agents and reactive oxygen species scavengers on ΔF/F response to dopamine. The addition of antioxidants (10 µM) did not inhibit the response of the (GT)$_6$-SWCNT sensor (10 ppm) to dopamine (10 µM). **a)** Fluorescence measurements of the (GT)$_6$-SWCNT 6 min after the addition of 10 µM of antioxidant. **b)** Fluorescence measurements of the (GT)$_6$-SWCNT 15 min after the addition of antioxidant (10 µM) followed 6 min later with addition of dopamine (10 µM). ΔF/F was measured after additive as the baseline. **c)** Ascorbic acid (10 µM) shows sensor response, with modest increase in response upon addition of dopamine (10 µM). For ascorbic acid only and **DA** only, ΔF/F was measured with no analyte as the baseline. For ascorbic acid + **DA**, ΔF/F was measured with ascorbic acid as the baseline.

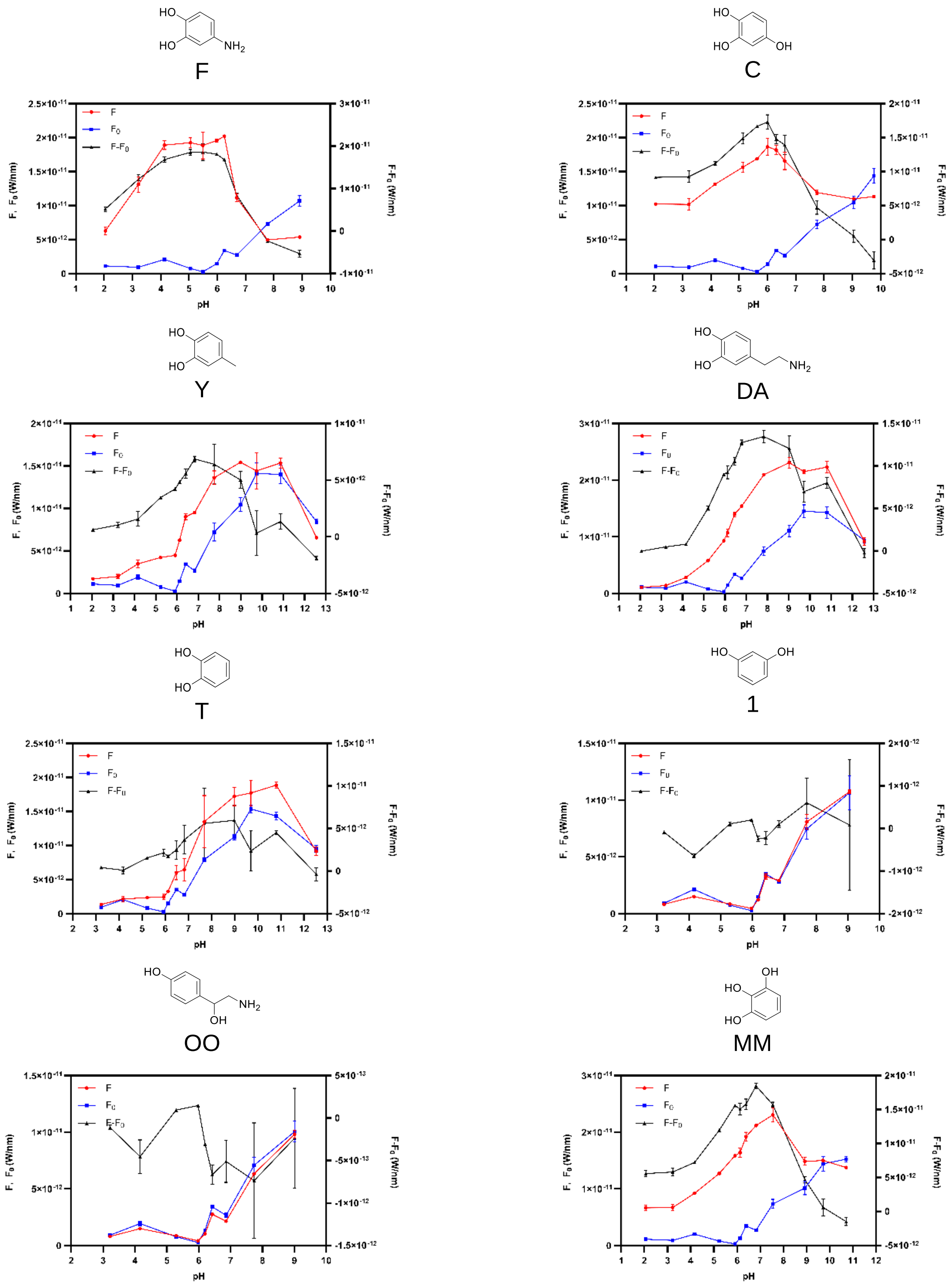

Figure S11. Eight compound optical responses measured over pH range of 2 – 13. Baseline fluorescence (blue, left axis, F_0), final fluorescence after addition of analyte (red, left axis, F), and change in fluorescence (black, right axis, $F-F_0$) are shown for each analyte over the pH range. Compound structures of the neutral version of the molecules are shown.

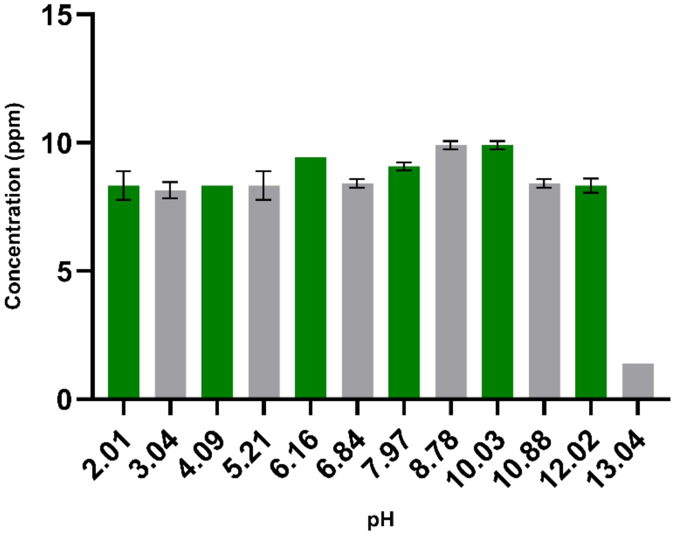

Figure S12. Stability of (GT)6-SWCNT after incubation at different pH's. SWCNT suspensions (10 µg/mL = 10 ppm) were titrated to the indicated pH and centrifuged at 20,000 RCF for 60 mins. Optical densities of the recovered supernatants were remeasured. SWCNT suspensions were generally stable between pH 2 – 12 and only noticeably precipitated at pH 13. All measurements (n = 3) were taken using a 1 mL solutions of 10 ppm $(GT)_6$-SWCNT suspension.

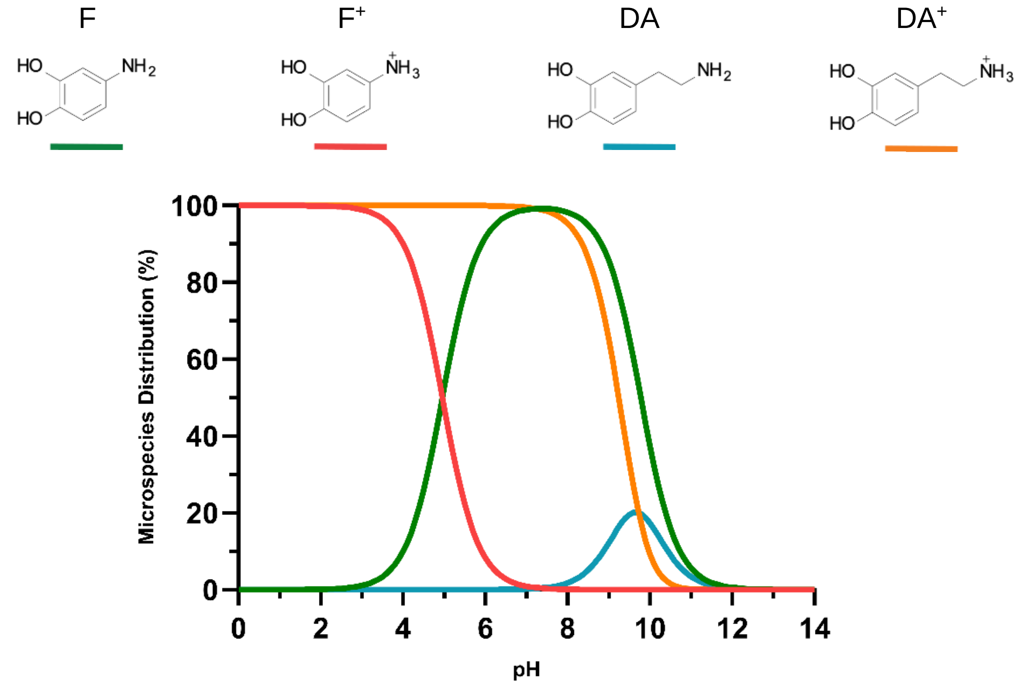

Figure S13. Microspecies distribution of F and DA. The microspecies vs. pH distribution plots were obtained from Chemaxon Chemicalize. Microspecies that were not of interest have been omitted. Neutral **F** and **DA** are shown in green and blue respectively. Cationic **F**$^{+}$ and **DA**$^{+}$ are shown in red and orange respectively.

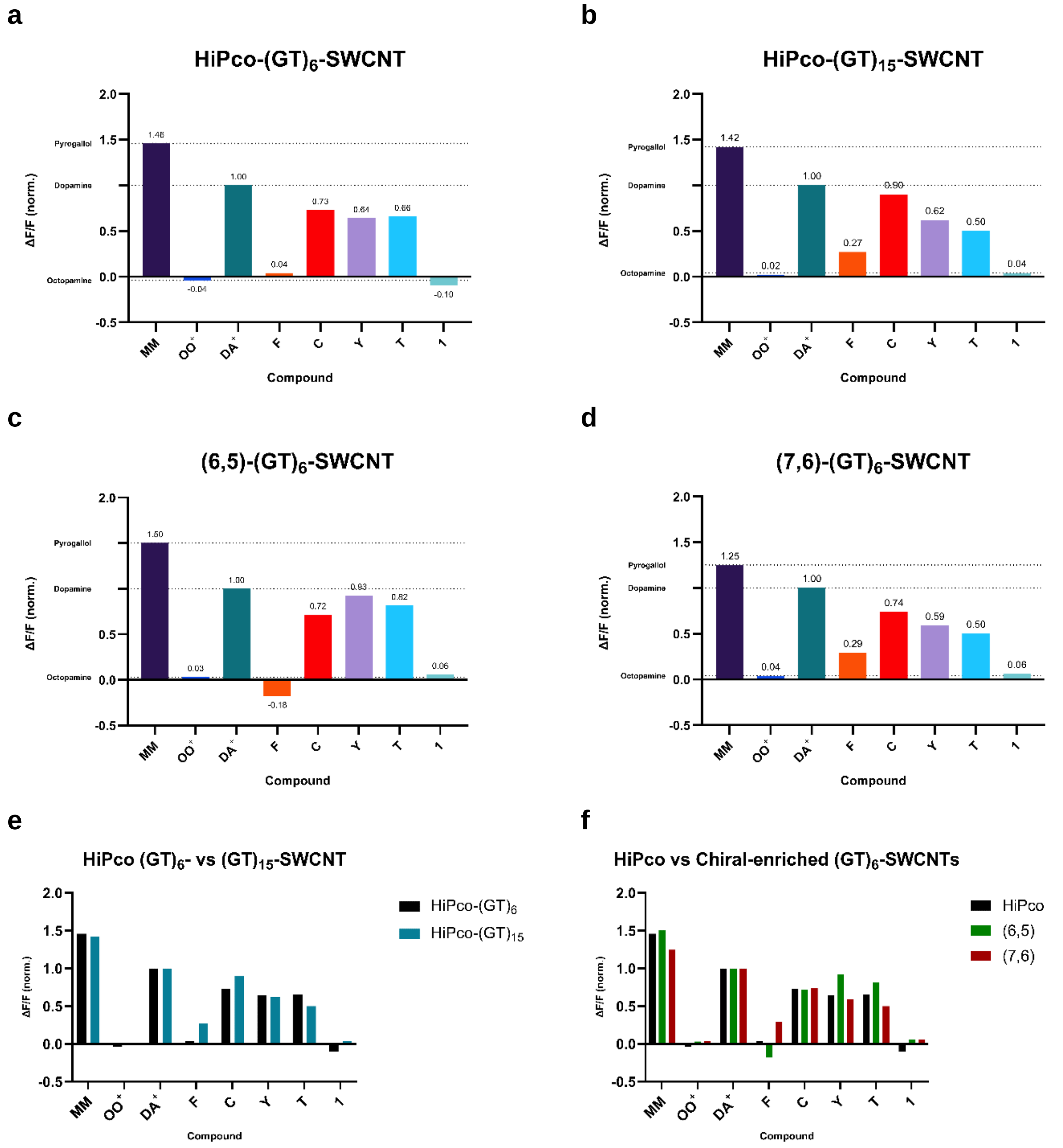

Figure S14. Comparison of normalized mean ΔF/F for 8 select compounds. Comparison using $(GT)_6$- vs $(GT)_{15}$-SWCNT ((**a**) vs. (**b**), respectively) and HiPco vs. (6,5)-enriched vs. (7,6)-enriched ((**a**) vs. (**c**) vs. (**d**), respectively). **e**), composite chart of (**a**) and (**b**), and **f**), composite chart of (**a**), (**c**) and (**d**) are presented to facilitate comparison. Composite figures show that the same general trends can be observed in both ssDNA sequences and chiralities suggesting that results can be generalized to $(GT)_N$ type systems conjugated to various chiralites. All readings were normalized to the response of dopamine = 1.00.

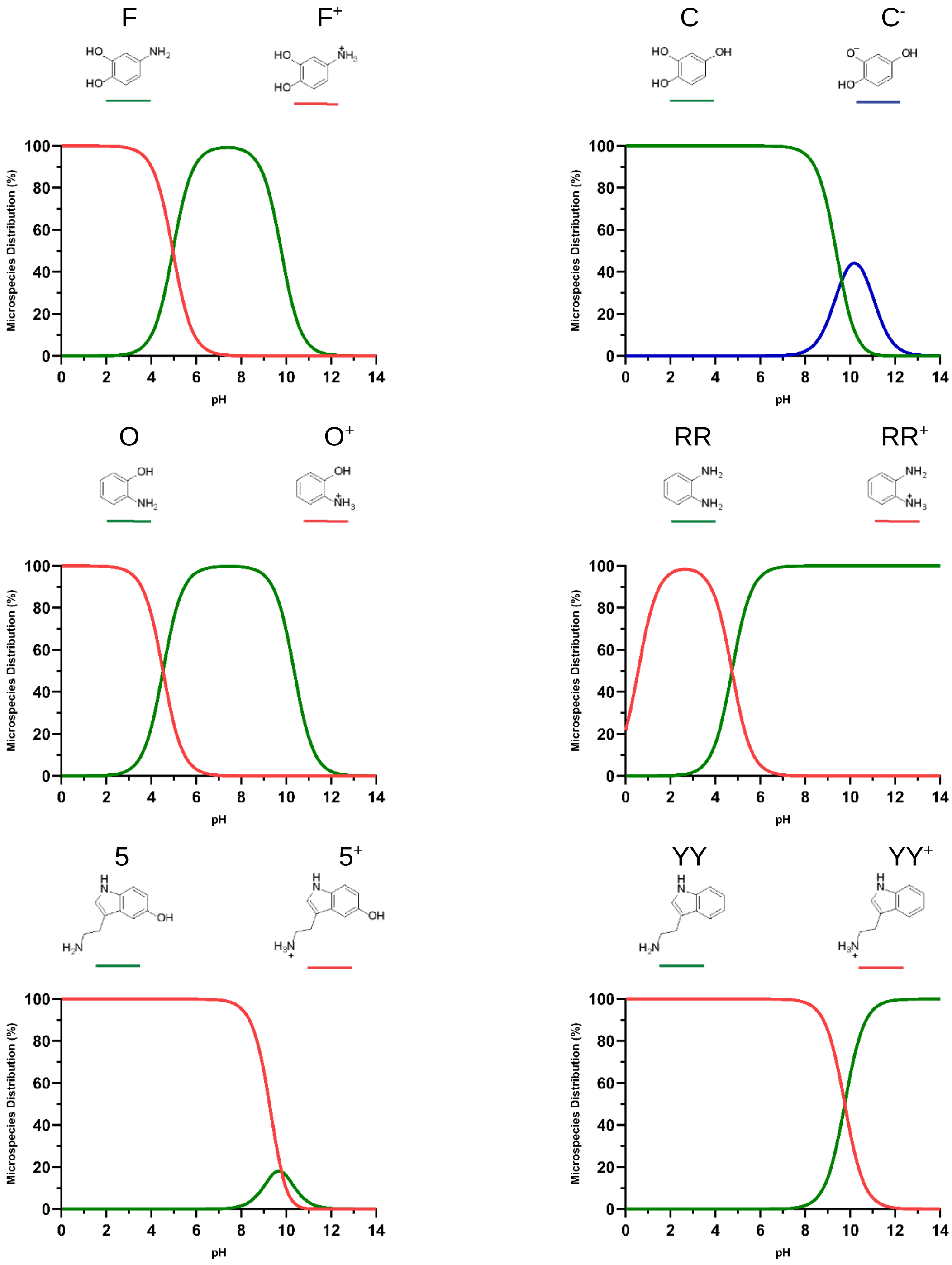

Figure S15. Protonation states of modeled molecules F, RR, YY, C, 5 and O at different pHs. The microspecies vs. pH distribution plots were obtained from Chemaxon Chemicalize. Microspecies that were not of interest have been omitted. Neutral compounds are shown in green, cations in red, and anions in blue.

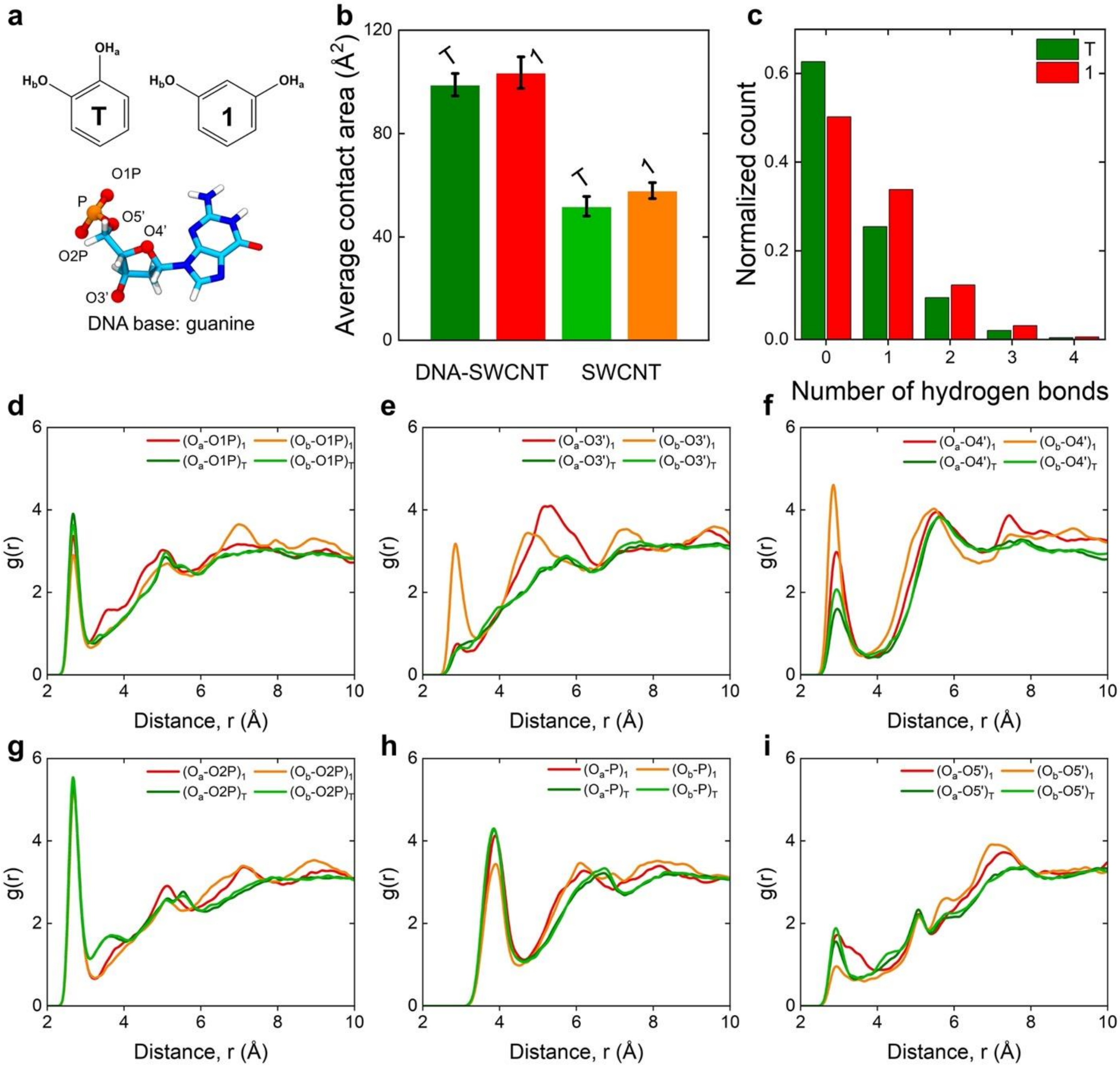

Figure S16. Investigation of molecular structure on analyte-sensor binding interaction for 1 and T a) Structure of T and 1**,** and ssDNA scaffold with labeled atoms whose interaction with **T** and **1** is quantified. **b)** The average contact areas of analyte molecules **T** and **1** with DNA-SWCNT conjugate and SWCNT surface. The contact areas were extracted from 6 µs trajectories and averaged over the six analyte molecules. **c)** The number of hydrogen bonds formed by **T** and **1**, with the DNA bases and the sugar and phosphate backbone. The cutoff radius for hydrogen bond formation was set at 3 Å and the cutoff angle was set at 20°. **(d-i)** interaction of labeled -OH groups on **T** and **1** with the labeled atoms on the ssDNA sugar and phosphate backbone, quantified in the form of radial distribution function, g(r). Interactions of **T** are shown in green hue and molecule 1 is shown in red hue.

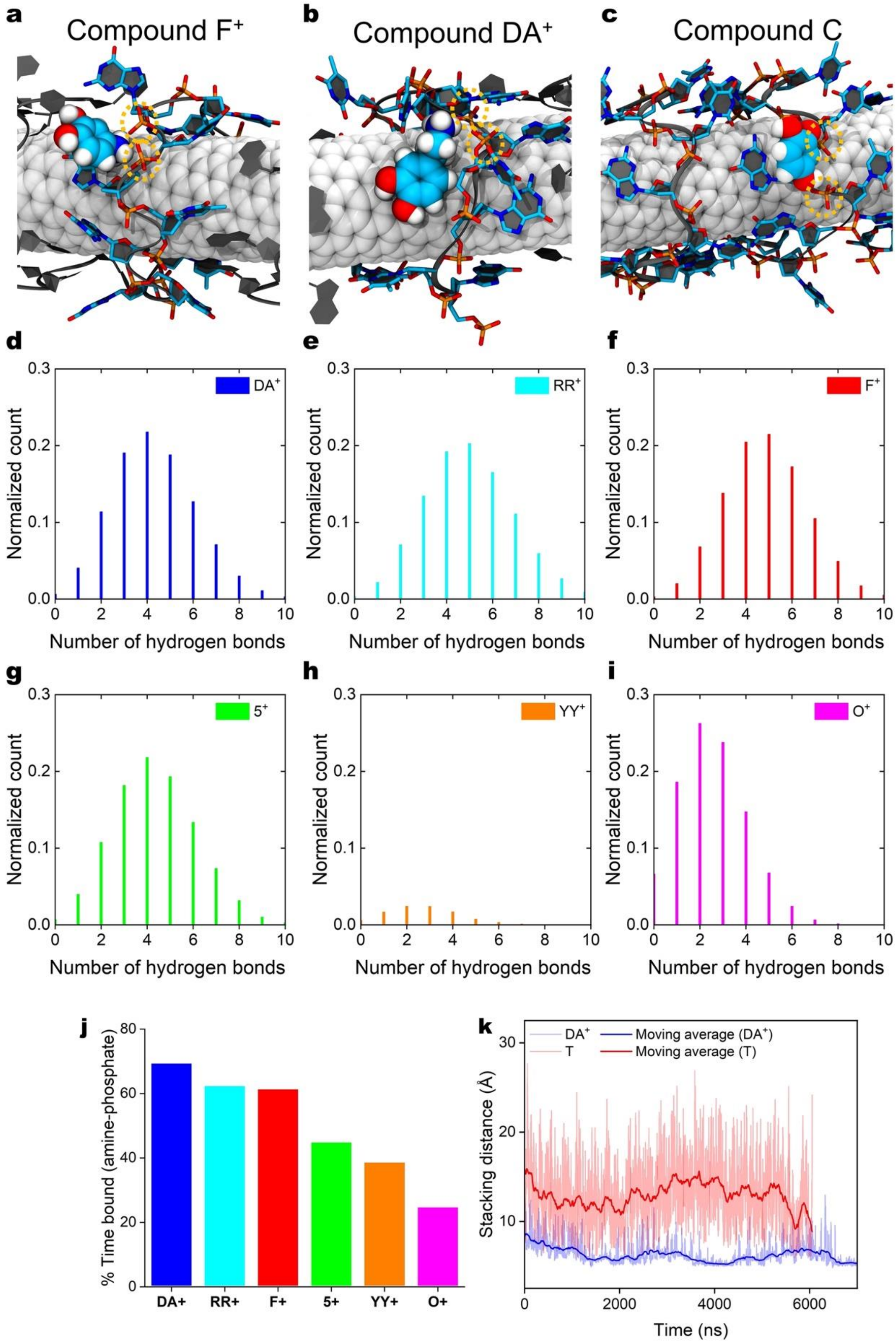
a
Compound F+
b
Compound DA+
c
Compound C
d
e
f
g
h
i
Normalized count
Number of hydrogen bonds
DA+
RR+
F+
5+
YY+
O+
j
% Time bound (amine-phosphate)
DA+
RR+
F+
5+
YY+
O+
k
Stacking distance (Å)
Time (ns)
Moving average (DA+)
T
Moving average (T)

Figure S17. Binding interactions between positively charged amine groups of analytes and negatively charged DNA phosphate backbone. a), b) The snapshots show **F**$^{+}$ and **DA**$^{+}$ in their predominant binding modes with their amine groups pointed towards the DNA phosphate backbone. **c)** In contrast, C is shown in its predominant binding mode, in which the -OH groups form transient hydrogen bonds with the DNA phosphate backbone atoms. SWCNT atoms are shown as white spheres, the $(GT)_6$ DNA strands are shown as dark grey ribbons, and the analyte molecules heavy atoms are shown as van der Waals spheres (C: cyan, N: blue, O: red). The orange dotted circles are used to highlight the phosphate groups participating in the interaction. **d-i)** Distributions of the number of hydrogen bonds formed between DNA (sugar-phosphate backbone and bases) and each of the positively charged analytes examined (**F**$^{+}$, **DA**$^{+}$, **RR**$^{+}$, **5**$^{+}$, **O**$^{+}$, and **YY**$^{+}$) calculated with VMD software. The cutoff radius for hydrogen bond formation was set to 3 Å and the cutoff angle was set to 20°. **j)** The plot shows the average percentage of time each of the analyte molecules spends with their amine groups interacting with the phosphate backbone in descending order. Only significant binding events (longer than 300 ns) were considered for this calculation. **k)** The plot shows analyte binding in terms of stacking distances for molecules **DA**$^{+}$ and **T**, over time, showing the dramatic effect that Coulombic interaction has on analyte-sensor binding stability

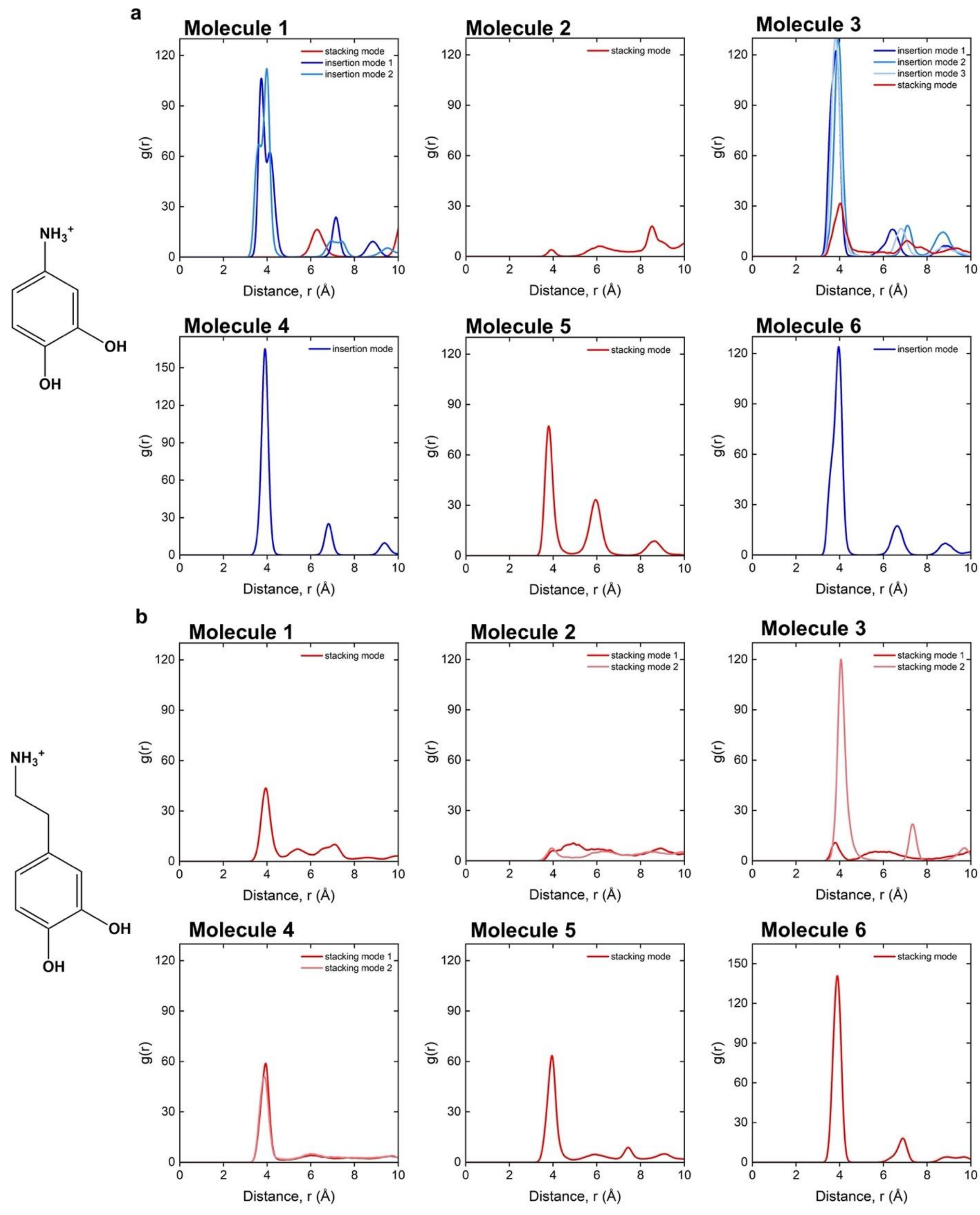

Figure S18. Radial distribution function g(r) analysis for interactions between the amino groups of F^+ and DA^+ and the phosphate groups on DNA. a) g(r) for the amino groups of analytes $\mathbf{F^+}$ and the phosphate groups on DNA in MD simulations. Separate plots are shown for each molecule, calculated from frames in which molecules exhibit the specified binding mode (insertion and stacking). The mode integer labels refer to g(r) calculated from frames of distinct binding events during the trajectory (some molecules unbind and rebind to the DNA-SWCNT

conjugate, and frames from each binding event are analyzed separately). Note that the g(r) shows a peak when **F+** inserts rather than stacks **b)** g(r) for the amino groups of analytes **DA^+** and the phosphate groups on DNA in MD simulations. Separate plots are shown for each molecule, calculated from frames in which molecules exhibit the specified binding mode (stacking only). The integer labels again refer to g(r) calculated from frames of distinct binding events. Note that g(r) achieves a maximum when DA+ stacks, and insertion interactions are not observed.

Table S1. Normalized compound ΔF/F values used in study for Figure 1b. Data arranged from high to low with **DA** and **OO** bracketing as in Figure 1.

**Compound**	**ΔF/F (Norm., mean)**	**Standard Deviation**	**Compound**	**ΔF/F (Norm., mean)**	**Standard Deviation**
DA	0.9330218069	0.05842679	K	0.0420560748	0.03738318
F	3.6651090343	0.11305450	W	0.0404984424	0.01641068
4	3.0809968847	0.37911953	U	0.0124610592	0.10294524
C	2.8037383178	0.16789948	WW	-0.0124610592	0.04416650
RR	2.7679127726	0.18493892	3	-0.0155763240	0.02573631
O	1.9252336449	0.08187577	6	-0.0389408100	0.00539580
MM	1.7398753894	0.05574932	J	-0.0436137072	0.01079159
UU	1.4890965732	0.15682659	DD	-0.0451713396	0.00269790
X	1.4672897196	0.53876296	S	-0.0498442368	0.03180775
KK	1.2788161994	0.05613963	CC	-0.0545171340	0.03805854
TT	1.0233644860	0.17440615	GG	-0.0560747664	0.02842412
2	0.8442367601	0.04996578	1	-0.0560747664	0.02472665
10	0.8348909657	0.22615728	ZZ	-0.0591900312	0.00539580
9	0.7367601246	0.03180775	8	-0.0623052960	0.01945482
D	0.5327102804	0.01401869	Q	-0.0638629283	0.01348949
7	0.5077881620	0.09137077	YY	-0.0669781931	0.01079159
EE	0.5062305296	0.01175987	P	-0.0669781931	0.02397945
Y	0.4968847352	0.07259302	FF	-0.0685358255	0.01641068
VV	0.4844236760	0.02573631	H	-0.0825545171	0.00539580
5	0.4766355140	0.01618739	M	-0.0887850467	0.02141391
SS	0.4485981308	0.03708997	G	-0.0887850467	0.00467290
LL	0.4221183801	0.08771282	PP	-0.0965732087	0.01348949
A	0.3504672897	0.03237478	JJ	-0.1261682243	0.00809370
I	0.3286604361	0.03974253	V	-0.1526479751	0.04214252
II	0.2757009346	0.03237478	Z	-0.1682242991	0.06491796
T	0.2087227414	0.03180775	BB	-0.1744548287	0.01888529
N	0.2087227414	0.02573631	AA	-0.2040498442	0.04240080
L	0.2087227414	0.11149863	XX	-0.2056074766	0.00809370
HH	0.1526479751	0.06098683	QQ	-0.2149532710	0.03527960
B	0.1355140187	0.04602270	E	-0.2881619938	0.02351973
R	0.0887850467	0.03649649	OO	-0.0996884735	0.00269790
NN	0.0420560748	0.03064224			

Table S2. ΔF/F and reduction potentials for Figure 2b. Data arranged from low to high as in figure.

Compound ID from Yamabe et al. (Based on studies of Pelizzetti et al.)	Compound ID from this study	Compound Name	One electron oxidation (V)	ΔF/F (Norm., mean)	Standard Deviation
1	EE	3-methoxybenzene-1,2-diol	1.18	0.50623053	0.01175987
4	7	4(1,1-dimethylethyl) benzene-1,2-diol	1.20	0.50778816	0.09137077
5	T	benzene-1,2-diol	1.25	0.20872274	0.03180775
6	A	4-chlorobenzene-1,2-diol	1.25	0.35046729	0.03237478
7	9	(-)-4-[l-hydroxy-2(methylamino)ethyl]benzene-l,2-diol (Adrenaline)	1.28	0.73676012	0.03180775
9	U	2,3-dihydroxybenzoic acid	1.36	0.01246106	0.10294524
10	B	3,4-dihydroxybenzoic acid	1.38	0.13551402	0.04602270
12	K	3,4-dihydroxybenzonitrile	1.43	0.04205607	0.03738318
13	HH	4-nitrobenzene-1,2-diol	1.46	0.15264798	0.06098683

Table S3. ΔF/F and Hammett values for Figure 2c. Data arranged from high to low as in figure.

Entry No.	Compound ID and Substituent	σ	ΔF/F (Norm., mean)	Standard Deviation
1	F - R-NH_2	-0.570	3.66510903	0.11305450
2	C - R-OH	-0.325	2.80373832	0.16789948
3	KK - R-OMe	-0.331	1.27881620	0.05613963
4	DA - dopamine	-0.129	0.93302181	0.05842679
5	9 - norepinephrine	-0.028	0.73676012	0.03180775
6	D - R-Et	-0.170	0.53271028	0.01401869
7	7 - *t*-butyl	-0.144	0.50778816	0.09137077
8	EE - R-OMe	0.029	0.50623053	0.01175987
9	Y - R-CH_3	-0.148	0.49688474	0.07259302
10	LL - R-Cl	0.378	0.42211838	0.08771282
11	A - R-Cl	0.200	0.35046729	0.03237478
12	I - R-ketone	0.455	0.32866044	0.03974253
13	N - R-F	0.129	0.20872274	0.02573631
14	T - catechol	0.000	0.20872274	0.03180775
15	HH - R-NO_2	0.984	0.15264798	0.06098683
16	B - R-COOH-para	0.523	0.13551402	0.04602270
17	K - R-CN	0.618	0.04205607	0.03738318
18	U - R-COOH-meta	0.366	0.01246106	0.10294524

Table S4. List of list of compounds, sources, calculated, and experimental values – see attached spreasheet document named “Table S4”

Table S5. Summary of simulations performed.

Analyte Type	Total Number of Atoms	Total Simulation Time (ns)
F	22255	6000
F^+	22324	6000
DA^+	22285	6000
C	22315	6000
C^-	22294	7000
Y	22306	6000
T	22303	6000
1	22315	6000
RR	22240	7000
YY^+	22216	7000
RR^+	22255	6000
5^+	22354	6000
O^+	22264	6000
O	22345	6000
